# Integrated RBF–EKF observer and MPC for simultaneous trajectory tracking and stability control of 4WID-EVs

**DOI:** 10.1038/s41598-026-53274-0

**Published:** 2026-05-21

**Authors:** Meng Dang, Chuanwei Zhang, Jianlong Wang, Yansong Feng

**Affiliations:** College of Mechanical Engineering, Xi’an University of Science and Technology, Xi’an, 710054 China

**Keywords:** Four-wheel-independent-drive electric vehicle, State estimation, Trajectory tracking, Model predictive control, Engineering, Mathematics and computing

## Abstract

Accurate trajectory tracking and stable motion control under extreme operating conditions remain significant challenges for Four-Wheel-Independent-Drive Electric Vehicles (4WID-EVs). To address these issues, this paper focuses on state estimation and trajectory tracking control, proposes novel algorithms and conducts comprehensive simulations and experiments. Firstly, a high-fidelity 7-Degree-of-Freedom (7-DOF) vehicle dynamics model is established and validated against a commercial CarSim model, providing a reliable platform for controller design. Secondly, to tackle the difficulty in directly measuring key states like the sideslip angle, a hybrid observer that fuses a Radial Basis Function Neural Network (RBFNN) with an Extended Kalman Filter (EKF) is proposed. The RBFNN, optimized via the control variates method, generates a "pseudo-sideslip angle," which is then fed as an observation into the EKF, significantly enhancing estimation accuracy. Thirdly, a Linear Time-Varying Model Predictive Control (LTV-MPC) based trajectory tracking controller is designed. The nonlinear vehicle model is linearized at each sampling point, transforming the optimal control problem into a Quadratic Programming (QP) problem. Crucially, explicit mathematical relationships between control variables and stability indices (sideslip angle, tire slip angle) are derived and embedded as stability constraints within the optimization framework, effectively coordinating tracking accuracy with vehicle stability. Finally, co-simulations (CarSim/Simulink) and Hardware-in-the-Loop (HIL) tests on a dSPACE platform are performed. Results demonstrate that the proposed RBF–EKF observer reduces the Root-Mean-Square Error (RMSE) of sideslip angle estimation by up to 30.43% compared to the standard EKF. Furthermore, the proposed Model Predictive Control (MPC) controller outperforms the traditional Preview Driver Model (PDM), reducing the maximum lateral tracking error and yaw angle error by 0.30 m and 3.11°, respectively, in high-speed double-lane-change scenarios, while ensuring all states remain within stability boundaries.

## Introduction

The automotive industry is undergoing a profound transformation, propelled by the dual disruptive trends of vehicle electrification and autonomous driving technologies^1^. While global economic growth and industrial advancement have fueled the sector’s expansion, they have concurrently exacerbated persistent challenges, including traffic accidents and congestion. Statistical analyses consistently identify human factors—such as failure to yield, unlicensed driving, and operational errors—as the predominant cause of traffic accidents worldwide^2^. In response to these safety and efficiency concerns, the development of intelligent driving systems has emerged as a critical global research imperative^3^.

Within this landscape, Connected and Automated Vehicles (CAVs) represent not merely the technological frontier but a strategic initiative aligned with the global consensus on enhancing road safety and traffic efficiency^4^. Studies have demonstrated that Connected and CAVs can fundamentally reduce human-error-induced traffic accidents and optimize traffic flow via collaborative perception and decision-making, thereby reshaping future mobility paradigms^5,6^. This pursuit is vigorously advanced by academia and industry globally, with significant progress reported in core technologies such as vehicle state estimation^7,8^, trajectory tracking control^9,10^, and multi-vehicle coordination^11^.

Among various vehicle platforms, 4WID-EVs offer unique advantages for trajectory tracking due to their independent actuation capabilities. Trajectory tracking control, a cornerstone of autonomous driving systems, is fundamental for accurate and safe path following, directly determining driving safety and operational intelligence^12,13^. This technology demands the system to achieve high-precision trajectory tracking while maintaining vehicle dynamic stability under diverse and complex driving conditions^14^. 4WID-EVs with their independently controllable actuators at each wheel, present an ideal platform for high-performance trajectory tracking, offering the potential to significantly enhance both tracking accuracy and active safety^15,16^. However, the efficacy of such control strategies is highly contingent upon the accuracy of key vehicle states, particularly the sideslip angle and yaw rate^17^. Often, due to limitations in sensor cost and direct measurement reliability, these critical states must be inferred via sophisticated estimation algorithms^18,19^. Consequently, research into integrated state estimation and trajectory tracking control for 4WID-EVs holds substantial theoretical and practical value^20,21^.

Extensive research has yielded a variety of trajectory tracking control strategies. In the past three years, a large number of high-impact studies on vehicle state estimation and trajectory tracking have been published in IEEE T-VT, T-ITS and IEEE Transactions on Intelligent Vehicles. For state estimation, hybrid estimation methods integrating Kalman filter and neural network have been widely studied, including UKF-informed neural network^22^, Transformer-enabled EKF^23^, adaptive strong tracking maximum correntropy criterion EKF^24^, Split-KalmanNet^25^ and FTEKFNet^26^. These methods improve estimation performance by fusing model-driven and data-driven ideas, but none of them design a dedicated "pseudo-sideslip angle" observation mechanism for EKF as proposed in this paper. For trajectory tracking control, advanced control strategies such as game theory-based MPC^27^ and distributed MPC^28^ have been applied to 4WID-EVs, but they fail to explicitly derive the mathematical relationship between control variables and stability indices (sideslip angle, tire slip angle) to realize dynamic stability constraint optimization. As systematically reviewed by Xiong et al.^29^, existing methods can be broadly categorized into: geometric model-based approaches (e.g., Pure Pursuit, Stanley), kinematic model-based methods employing feedback linearization, and dynamic model-based advanced control techniques, including Proportional-Integral-Derivative (PID) control, MPC, Active Disturbance Rejection Control (ADRC), and Sliding Mode Control (SMC). Inspired by human driving behavior, preview control constitutes another significant research direction. MacAdam^30^ pioneered the foundational preview driver model, which was later advanced by Li et al.^31^ through an adaptive preview strategy for extreme conditions, enhancing system robustness. A predominant limitation of such methods, however, is their reliance on empirical rules, often lacking a rigorous theoretical framework to guarantee universal optimality.

MPC has garnered considerable attention in trajectory tracking due to its explicit capability to handle multivariable coupling and state/input constraints^32,33^. Its core principle involves forward prediction based on a dynamic model and solving for an optimal control sequence over a finite horizon through rolling optimization. Falcone et al.^34^ pioneered the application of MPC for front steering control of autonomous vehicles, demonstrating its constraint-handling capability and tracking accuracy via real vehicle tests. Ji et al.^35^ designed a hierarchical framework integrating planning and tracking, employing Nonlinear Model Predictive Control (NMPC) as the tracker, which exhibited exceptional dynamic performance. Nonetheless, NMPC is hampered by inherent challenges, including high online computational complexity and difficulties in ensuring real-time convergence, posing significant obstacles for deployment on vehicle platforms demanding stringent real-time performance.

The emergence of 4WID-EVs, with their flexible actuation potential, introduces distinct research opportunities and challenges. While providing additional control degrees of freedom for coordinating trajectory tracking and stability control, the over-actuated nature and strong tire force nonlinearities of 4WID-EVs present significant control challenges^36^. Hierarchical control architectures have consequently become a mainstream solution. Zhang et al.^37^ developed a two-layer structure where an upper-layer MPC computes the desired front steering angle and yaw moment, and a lower layer performs optimal torque distribution. While structurally clear, this approach often lacks explicit, mechanistic handling of time-varying constraints on critical stability parameters. Falcone et al.^38^ developed an MPC controller coordinating steering and braking based on a simplified nonlinear model, demonstrating practical potential albeit with possible sacrifices in control fidelity. To balance the computational burden and performance of NMPC, Wu et al.^39^ adopted a LTV-MPC approach, effectively reducing complexity, though potential linearization errors in highly nonlinear regions remain a concern. Similarly, Guo et al.^40^ utilized an LTV model fused with Linear Matrix Inequalities to design a robust controller, enhancing the handling of model uncertainties. Wu et al.^41^ proposed a decoupled hierarchical scheme employing MPC for lateral and SMC for longitudinal control, a design that combines methodological strengths but may increase inter-controller coordination complexity.

Despite these advancements, a critical gap persists in existing LTV-MPC frameworks, particularly concerning the treatment of stability constraints. Current methods often apply empirical, fixed boundaries to key states like the sideslip angle, failing to establish an explicit, mechanistic mathematical relationship between these stability criteria and the control inputs. This limitation inherently restricts the controller’s ability to achieve optimal coordination between trajectory tracking and dynamic stability, especially under extreme operating conditions where such coordination is paramount.

To address this gap, this paper proposes an enhanced LTV-MPC strategy for 4WID-EVs, with its key novelty lying in the explicit, model-based integration of dynamic stability constraints. The main contributions are threefold: First, a hybrid state observer, which innovatively combines a Radial Basis Function (RBF) neural network with an EKF, is designed to achieve high-fidelity estimation of critical states. Different from conventional hybrid observers, the RBFNN is optimized by the control variates method to generate a dedicated "pseudo-sideslip angle" as the observation of EKF, which solves the problem that the sideslip angle cannot be directly measured and breaks through the accuracy limitation of single EKF or RBF observer. Second, and most importantly, the LTV-MPC controller is formulated with stability constraints derived directly from vehicle dynamics principles. Instead of using empirical fixed stability boundaries, this paper explicitly deduces the mathematical relationship between control variables and stability indices (sideslip angle, tire slip angle), and embeds dynamic stability constraints into the optimization framework to actively coordinate tracking accuracy and vehicle stability. Finally, the real-time performance, effectiveness, and robustness of the proposed integrated strategy are rigorously validated under typical extreme maneuvers (e.g., double lane change on low-friction surfaces) using a dSPACE HIL platform, providing a comprehensive theoretical and experimental basis for practical engineering applications.

The remainder of this paper is organized as follows: Section "Distributed drive electric vehicle dynamic modeling" details the establishment of the high-fidelity vehicle dynamics model. Section "Vehicle state estimation using a hybrid RBF–EKF observer" introduces the design of the RBF–EKF hybrid observer. Section "Trajectory tracking control strategy for 4WID-EVs" elaborates on the design of the stability-constrained LTV-MPC controller. Section "Hardware-in-the-loop experiment for trajectory tracking" presents both simulation and HIL experimental results. Finally, Section "Conclusion" concludes the paper and discusses future work.

## Distributed drive electric vehicle dynamic modeling

### Vehicle dynamic model

**Fig. 1:**
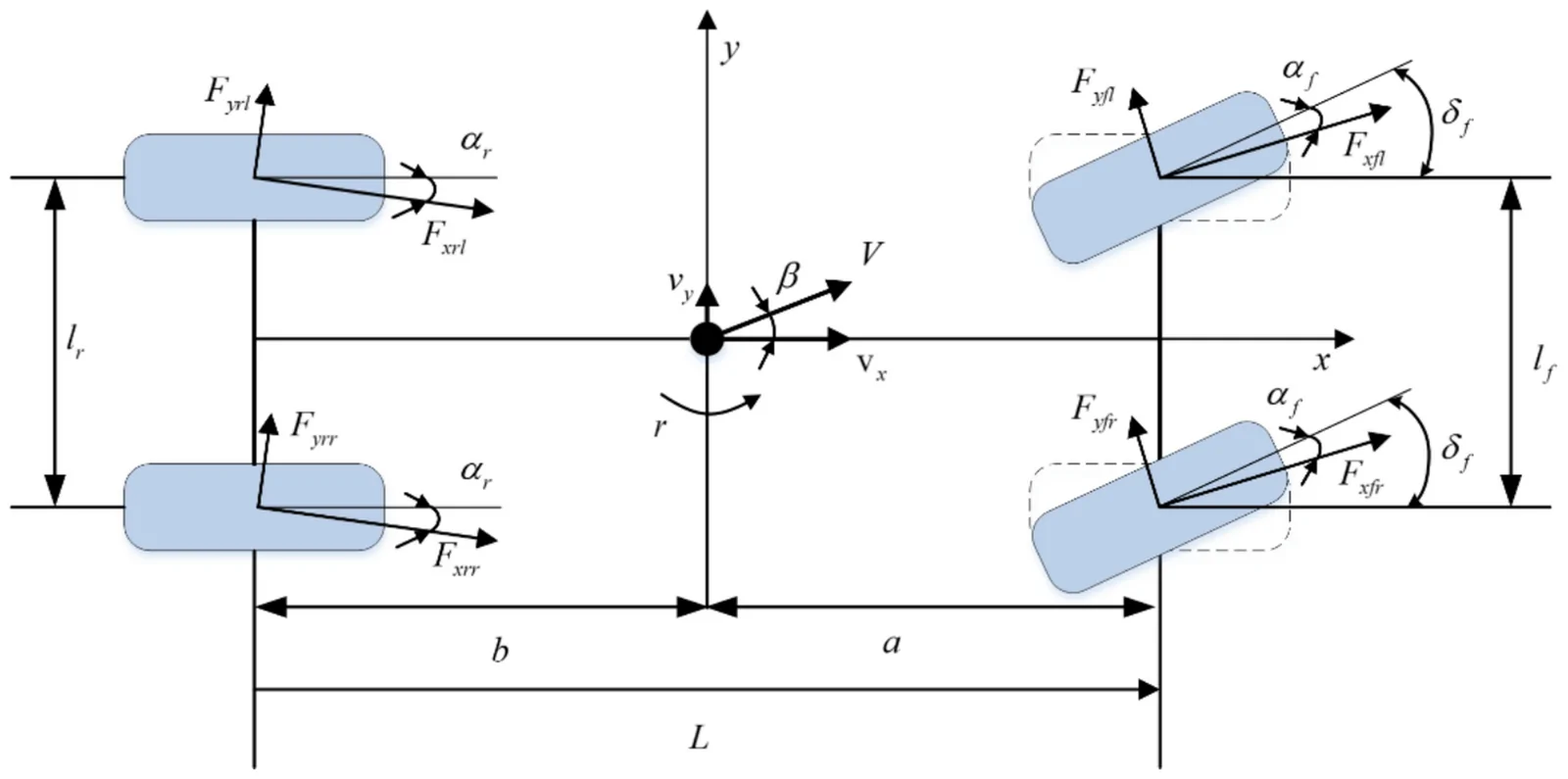
Schematic of the force analysis for the 7-DOF vehicle model.

This study focuses on analyzing the trajectory tracking control and overall stability of 4WID-EVs, while considerations such as energy economy and ride comfort are beyond its scope. The 4WID-EV represents a highly complex nonlinear system characterized by strong coupling and intricate interactions among its various attributes^42^. To capture the essential dynamics, a 7-DOF vehicle dynamics model is established within the body-fixed coordinate system. This model incorporates three DOFs for the vehicle body (longitudinal, lateral, and yaw motion) and four DOFs for the rotational dynamics of each wheel. Figure 1 illustrates the schematic of the vehicle force analysis.

Accounting for load transfer and torque distribution during vehicle motion, the dynamic relationships are established among the vehicle’s longitudinal motion, lateral motion, yaw motion, the torque of each wheel, and the steering wheel angle.

The longitudinal dynamics equation is given by:

1 $$\left\{\begin{array}{l}{a}_{x}={\dot{v}}_{x}-r{v}_{y}\\ {a}_{x}=\frac{1}{m}\left[\left({F}_{xfl}+{F}_{xfr}\right)\mathrm{cos}\,\delta -\left({F}_{yfl}+{F}_{yfr}\right)\mathrm{sin}\,\delta +{F}_{xrl}+{F}_{xrr}\right]\end{array}\right.$$

where ${a}_{x}$ is the absolute longitudinal acceleration ($\mathrm{m}/{\mathrm{s}}^{2}$), ${v}_{x}$ is the longitudinal velocity at center of mass (CoG) ($\mathrm{m}/\mathrm{s}$), ${v}_{y}$ is the lateral velocity at CoG ($\mathrm{m}/\mathrm{s}$), *r* is the yaw rate ($\mathrm{r}\mathrm{a}\mathrm{d}/\mathrm{s}$), *m* is the total vehicle mass ($\mathrm{k}\mathrm{g}$), ${F}_{xij}$ is the longitudinal tire force ($\mathrm{N}$), ${F}_{yij}$ is the lateral tire force ($\mathrm{N}$), *δ* is the front wheel steering angle ($\mathrm{r}\mathrm{a}\mathrm{d}$), subscripts *fl*, *fr*, *rl*, *rr* denote front-left, front-right, rear-left, rear-right wheels, respectively.

The lateral dynamics equation is expressed as:

2 $$\left\{\begin{array}{l}{a}_{y}={\dot{v}}_{y}+r{v}_{x}\\ {a}_{y}=\frac{1}{m}\left[\left({F}_{xfl}+{F}_{xfr}\right)\mathrm{sin}\,\delta +\left({F}_{yfl}+{F}_{yfr}\right)\mathrm{cos}\,\delta +{F}_{yrl}+{F}_{yrr}\right]\end{array}\right.$$

where ${a}_{y}$ is the absolute lateral acceleration ($\mathrm{m}/{\mathrm{s}}^{2}$), and other variables are defined the same as in Eq. (1)

The yaw dynamics equation is expressed as:

3 $$\left\{\begin{array}{l}{M}_{z}={I}_{zz}\dot{r}\\ {M}_{z}=a\left({F}_{xfl}+{F}_{xfr}\right)\mathrm{sin}\,\delta +a\left({F}_{yfl}+{F}_{yfr}\right)\mathrm{cos}\,\delta -b\left({F}_{yrl}+{F}_{yrr}\right)\\ -\frac{{l}_{f}}{2}\left({F}_{xfl}-{F}_{xfr}\right)\mathrm{cos}\,\delta +\frac{{l}_{f}}{2}\left({F}_{yfl}-{F}_{yfr}\right)\mathrm{sin}\,\delta -\frac{{l}_{r}}{2}\left({F}_{xrl}+{F}_{xrr}\right)\end{array}\right.$$

where ${M}_{z}$ is the external yaw moment ($\mathrm{N}\,\mathrm{m}$), ${I}_{zz}$ is the yaw moment of inertia ($\mathrm{kg}\,{\mathrm{m}}^{2}$), *a* is the distance from CoG to front axle (*m*), *b* is the distance from CoG to rear axle (*m*), ${l}_{f}$ is the front track width (*m*), ${l}_{r}$ is the rear track width (*m*), and other variables are defined the same as above.

The dynamic model incorporates key parameters where ${F}_{xij}$ represents the longitudinal force generated by each wheel, with subscripts *ij* denoting wheel positions: front-left (*fl*), front-right (*fr*), rear-left (*rl*), and rear-right (*rr*). The vehicle’s total mass is denoted by *m*, while ${v}_{x}$ and ${v}_{y}$ represent the longitudinal and lateral velocities at the CoG, respectively. The yaw rate is given by *r*. Geometric parameters include *a* and *b*, representing the distances from the CoG to the front and rear axles, and ${I}_{zz}$ denoting the vehicle’s moment of inertia about the vertical axis. The front wheel steering angle, assumed identical for both front wheels, is denoted by *δ*. The vehicle’s sideslip angle at the CoG is *β*, and *V* is the resultant velocity at the CoG. Acceleration terms include absolute longitudinal acceleration ${a}_{x}$, absolute lateral acceleration ${a}_{y}$, and the corresponding accelerations at the CoG: longitudinal acceleration ${\dot{v}}_{x}$ and lateral acceleration ${\dot{v}}_{x}$.

### Wheel dynamics

**Fig. 2:**
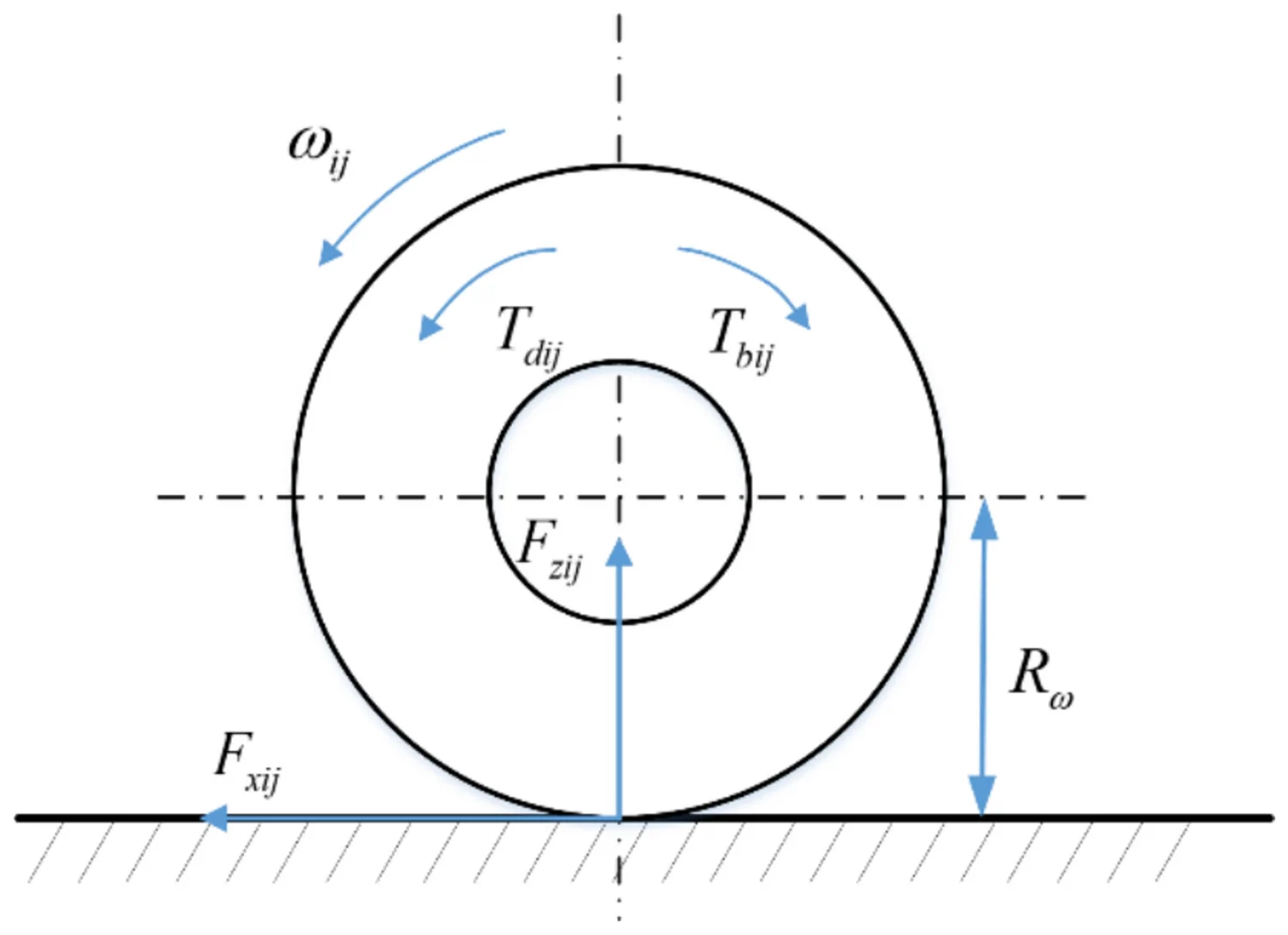
Schematic diagram of the single driven wheel dynamics.

In a four-wheel-independent-drive electric vehicle, each wheel can function as a driven wheel. Figure 2 illustrates the dynamic principles of the tire during rolling, where the rolling resistance and resistance moment due to tire and road deformation are neglected. Among the four wheels, ${T}_{d,ij}$ represents the driving torque applied to a specific wheel by its drive motor, while ${T}_{b,ij}$ denotes the braking torque generated by the brake caliper or disc that opposes wheel rotation. Additionally, ${T}_{x,ij}$ is the reaction torque from the ground resulting from friction as the tire rolls forward, and the rotational speed of the wheel is denoted by ${\omega}_{ij}$. The force ${F}_{z,ij}$ represents the vertical load on the tire, which corresponds to the portion of the vehicle mass supported by that tire. The subscript *ij=fl,fr,rl,rr* indicates the four wheel positions: front-left, front-right, rear-left, and rear-right. The effective rolling radius of the wheel is denoted as ${R}_{e}$, and the dynamic equation is expressed as follows:

4 $$G\left\{\begin{array}{l}{I}_{w}{\dot{\omega }}_{fl}=-{R}_{w}{F}_{xfl}+{T}_{dfl}-{T}_{bfl}\\ {I}_{w}{\dot{\omega }}_{fr}=-{R}_{w}{F}_{xfr}+{T}_{dfr}-{T}_{bfr}\\ {I}_{w}{\dot{\omega }}_{rl}=-{R}_{w}{F}_{xrl}+{T}_{drl}-{T}_{brl}\\ {I}_{w}{\dot{\omega }}_{rr}=-{R}_{w}{F}_{xrr}+{T}_{drr}-{T}_{brr}\end{array}\right.$$

During driving maneuvers such as acceleration, deceleration, and cornering, the tires experience complex load variations due to dynamic load transfer. During deceleration, inertial forces increase the vertical load on the front wheels and decrease the load on the rear wheels. Similarly, during cornering, centrifugal force causes an increase in load on the outer wheels and a decrease on the inner wheels^43^. The vertical load on each wheel can be calculated using Eq. (5):

5 $$\left\{\begin{array}{l}{F}_{zfl}=mg\frac{b}{2L}-m{a}_{x}\frac{h}{2L}-m{a}_{y}\frac{hb}{{l}_{f}L}\\ {F}_{zfr}=mg\frac{b}{2L}-m{a}_{x}\frac{h}{2L}+m{a}_{y}\frac{hb}{{l}_{f}L}\\ {F}_{zrl}=mg\frac{a}{2L}+m{a}_{x}\frac{h}{2L}-m{a}_{y}\frac{ha}{{l}_{r}L}\\ {F}_{zrr}=mg\frac{a}{2L}+m{a}_{x}\frac{h}{2L}+m{a}_{y}\frac{ha}{{l}_{r}L}\end{array}\right.$$

In these equations, ${F}_{zij}$ represents the vertical load on each wheel, with the subscript *ij* indicating the wheel position: front-left (*fl* ), front-right (*fr*), rear-left (*rl*), and rear-right (*rr*); *h* is the height of the center of mass; *L* is the wheelbase, calculated as the sum of the distances from the center of mass to the front axle *a* and to the rear axle *b* (i.e., *L=a+b*); ${d}_{f}$ and ${d}_{r}$ are the front and rear track widths, respectively; *μ* is the road friction coefficient; and ${a}_{x}$ and ${a}_{y}$ are the longitudinal and lateral accelerations, respectively.

### Tire slip angle

The lateral elasticity of tires generates a lateral reaction force at the contact patch, known as the cornering force, when the vehicle is subjected to banked roads, crosswinds, or steering maneuvers. This force causes a deviation between the wheel’s actual travel direction and its geometric plane (the plane perpendicular to the rotation axis), a phenomenon referred to as tire slip^44^. The angle formed between the wheel’s travel direction and its plane is defined as the slip angle α, whose magnitude directly correlates with the lateral force magnitude—this relationship defines the tire’s cornering characteristics. The slip angles for the four wheels are calculated using Eq. (6):

6 $$\left\{\begin{array}{l}{\alpha}_{fl}=\delta -{\mathrm{tan}}^{-1}\frac{{v}_{y}+ar}{{v}_{x}-\frac{{l}_{f}}{2}r}\\ {\alpha}_{fr}=\delta -{\mathrm{tan}}^{-1}\frac{{v}_{y}+ar}{{v}_{x}+\frac{{l}_{f}}{2}r}\\ {\alpha}_{rl}=-{\mathrm{tan}}^{-1}\frac{{v}_{y}-br}{{v}_{x}-\frac{{l}_{r}}{2}r}\\ {\alpha}_{rl}=-{\mathrm{tan}}^{-1}\frac{{v}_{y}-br}{{v}_{x}-\frac{{l}_{r}}{2}r}\end{array}\right.$$

where ${\alpha}_{ij}$ denotes the tire slip angle with subscripts $ij=\{fl,fr,rl,rr\}$ indicating front-left, front-right, rear-left, and rear-right wheels respectively; *δ* is the steering angle of the front wheels (assumed identical for both sides in this model); *r* represents the yaw rate; ${v}_{x}$ and ${v}_{y}$ are the longitudinal and lateral velocities at the center of mass; *a* and *b* are the distances from the center of mass to the front and rear axles; and ${l}_{f}$, ${l}_{r}$ represent the front and rear track widths respectively.

### Wheel slip ratio

During curvilinear motion, the longitudinal velocity of each wheel center in the tire coordinate system is influenced by the vehicle’s yaw motion and can be described by Eq. (7):

7 $$\left\{\begin{array}{l}{v}_{xfl}=\left({v}_{x}-\frac{{l}_{f}}{2}r\right)\mathrm{cos}\,\delta +\left({v}_{y}+ar\right)sin\delta \\ {v}_{xfr}=\left({v}_{x}+\frac{{l}_{f}}{2}r\right)\mathrm{cos}\,\delta +\left({v}_{y}+ar\right)sin\delta \\ {v}_{xrl}={v}_{x}-\frac{{l}_{r}}{2}r\\ {v}_{xrr}={v}_{x}+\frac{{l}_{r}}{2}r\end{array}\right.$$

The wheel slip ratio plays a critical role in determining the longitudinal tire force. This ratio, bounded between 0 and 1, quantifies the degree of wheel slip from pure rolling to complete sliding. The slip ratio is calculated using Eq. (8), where a value of 0 indicates pure rolling without slip, while a value of 1 corresponds to complete wheel sliding during braking or other extreme conditions:

8 $${\lambda}_{ij}=\frac{{\omega}_{ij}{R}_{w}-{v}_{xij}}{{v}_{xij}}$$

where ${\lambda}_{ij}$ denotes the slip ratio of each wheel; ${\omega}_{ij}$ represents the wheel’s rotational speed; ${v}_{xij}$ indicates the longitudinal velocity of each wheel center in the wheel coordinate system; and ${R}_{w}$ is the effective rolling radius of the wheel.

### In-wheel motor model

The in-wheel motor plays a central role in the drive system, directly determining the vehicle’s dynamic performance and influencing overall vehicle characteristics. Among the four main types of hub motors—permanent magnet brushless motors, permanent magnet synchronous motors, induction motors, and switched reluctance motors—the permanent magnet brushless DC motor (PMBLDC) stands out due to its significant advantages in weight and volume reduction, excellent speed regulation capability, low torque ripple, and high energy efficiency, ensuring high power output and stable operation^45^.

Accordingly, a PMBLDC motor model was developed in Simulink and integrated with the previously established wheel model. Compared to mechanical systems, the brushless DC motor exhibits significantly faster torque response. Therefore, the relationship between the actual electromagnetic torque and the desired torque in the PMBLDC motor model is simplified as a second-order system, represented by the transfer function in Eq. (9):

9 $$G\left(s\right)=\frac{{T}_{a}}{{T}_{d}}=\frac{1}{1+2\xi s+2{\xi }^{2}{s}^{2}}$$

where *ξ* is the damping ratio characterizing the motor’s dynamic properties, and *s* represents the complex frequency variable in the Laplace domain.

## Vehicle state estimation using a hybrid RBF–EKF observer

Accurate knowledge of vehicle states and parameters is crucial for vehicle system management and control, as it directly impacts trajectory tracking capability and driving safety. State estimation methods provide a cost-effective means to obtain dynamic vehicle information, which is particularly important for nonlinear vehicle systems. This paper proposes a novel observer that integrates a RBF neural network with an EKF to estimate the vehicle sideslip angle and yaw rate. This method effectively combines the advantages of both approaches. Considering the impact of input data types and sensor measurability, and referencing the nonlinear vehicle model, a mapping relationship between system inputs and outputs is established. Based on this mapping, an RBF neural network estimator is designed. To reduce noise influence and improve algorithm reliability, this neural network is combined with the Kalman filter.

### RBF neural network sideslip angle estimator

RBF neural networks have garnered significant attention due to their strong generalization capability, simple network structure, and fast training speed. Furthermore, relevant research has confirmed that RBF neural networks can approximate any nonlinear function with arbitrary accuracy^46^.

Parameter Selection Basis of RBF Neural Network: The number of hidden layer neurons is determined within the universal range of 15–25 for vehicle state estimation by the control variates method. Considering the trade-off between nonlinear approximation accuracy and online computational efficiency, 20 hidden layer neurons are selected as the optimal value. The kernel function adopts the standard Gaussian radial basis function, which is the most suitable kernel for vehicle dynamics nonlinear fitting. The kernel width parameters are set according to the classical configuration of RBF neural networks, which ensures the stability and convergence of network training.

Feature Parameter Selection: The factors considered for the RBF algorithm input selection include: the minimal number of input data types, and the selection of data measurable by vehicle sensors. From Eqs. (3) and (4), it can be derived that:

10 $$\beta =f(r,{V}_{x},{a}_{y},\delta )$$

Therefore, the following data types are selected as inputs for the neural network: yaw rate (*r*), longitudinal velocity (${V}_{x}$), steering wheel angle (${\delta}_{s}$)—since the front wheel angle is difficult to measure directly, the steering wheel angle, which has a corresponding relationship with it, is chosen—and lateral acceleration (${a}_{y}$).

RBF Neural Network Estimator: The RBF neural network consists of three layers: the input layer, the radial basis function layer (hidden layer), and the output layer. Input Layer: Data is fed into the network here.

Hidden Layer: Neurons using radial basis functions as their activation mechanism constitute the hidden layer. These computational elements are collectively termed hidden nodes. Output Layer: The network output is realized using a weighted sum function.

### Extended Kalman filter estimator

The Extended Kalman Filter is an adaptation of the basic Kalman Filter for nonlinear systems. It linearizes the state equations via Taylor expansion around the current estimate, ignoring second-order and higher terms, i.e., by calculating the Jacobian matrices of the state transition equations. This results in a linear approximation model, to which the standard KF algorithm is then applied to estimate the desired states. The primary difference between EKF and KF lies in the EKF’s computation of the Jacobian matrices for linearization.

The EKF procedure is summarized as follows:

1. State Prediction:
  11 *X(k+1|k)=f[X(k),U(k)]+W(k)*
2. Measurement Prediction:
  12 *Y(k+1|k)=h[X(k+1|k),U(k)]+R(k)*
3. Prediction of the Error Covariance:
  13 $$P(k+1|k)=A(k+1|k)P(k){A}^{T}+Q(k+1)$$
4. Calculation of the Kalman Gain:
  14 $$K(k+1)=P(k+1|k){H}^{T}[H(k+1)P(k+1|k){H}^{T}+R(k+1){]}^{-1}$$
5. State Estimate Update using Measurement:
  15 *X(k+1|k+1)=X(k+1|k)+K(k+1)[Y(k+1)-Y(k+1|k)]*
6. Error Covariance Update:
  16 *P(k+1)=[I-K(k+1)H]P(k+1|k)*

### Hybrid RBF–EKF vehicle state observer

**Fig. 3:**
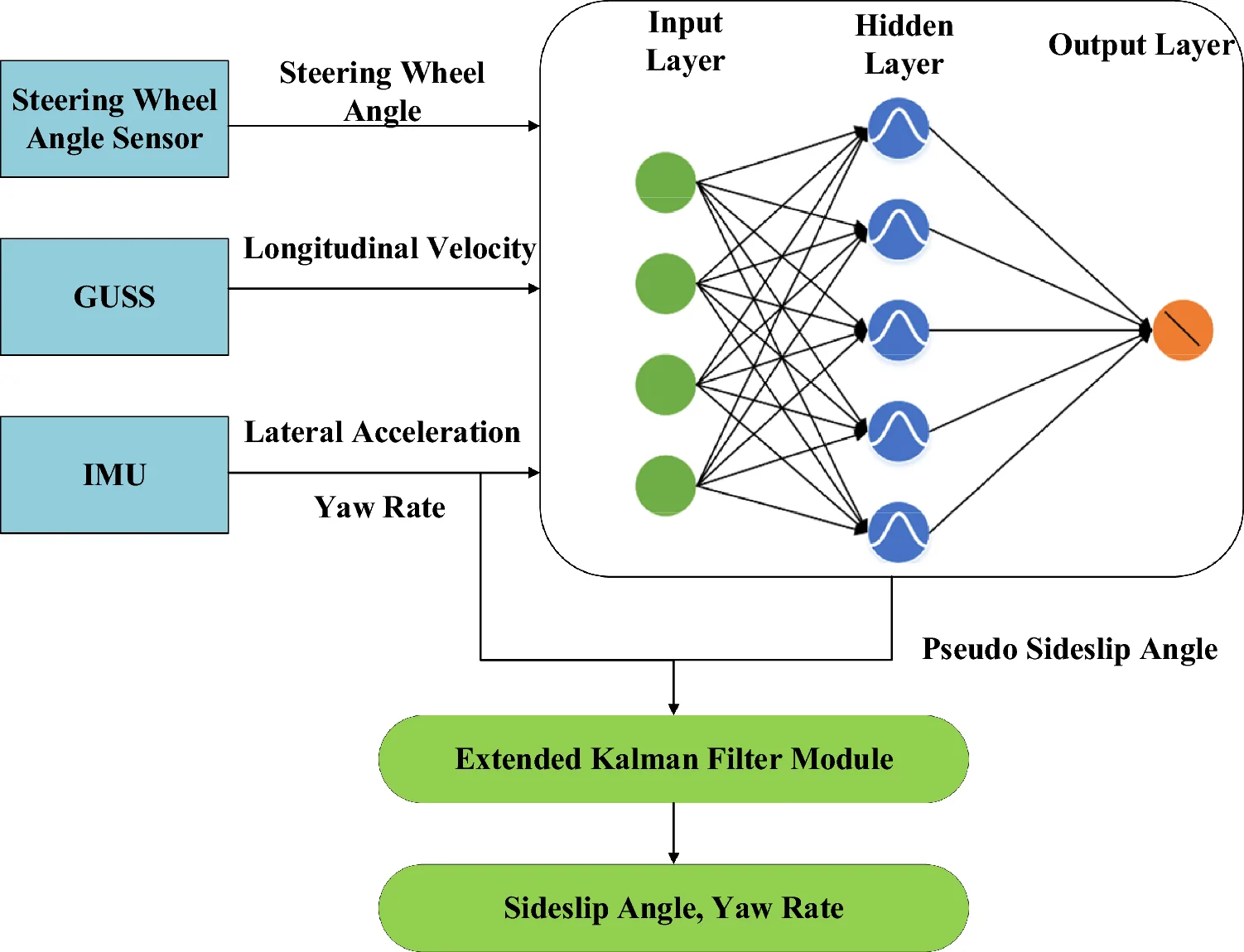
Structure of the vehicle state observer.

The structure of the proposed observer is illustrated in Fig. 3. The unique design logic of this hybrid observer lies in the generation and fusion mechanism of the "pseudo-sideslip angle", which is essentially different from the existing RBF–EKF fusion structures. The first stage is an RBF neural network-based estimator, which generates an initial estimate of the sideslip angle (referred to as the "pseudo-sideslip angle"). The RBFNN is optimized by the control variates method with measurable sensor signals (steering wheel angle, longitudinal velocity, lateral acceleration, yaw rate) as inputs, ensuring the pseudo-sideslip angle fits the nonlinear dynamic characteristics of 4WID-EVs. The inputs to this stage are the steering wheel angle measured by a sensor, the longitudinal velocity obtained from a GNSS receiver, and the lateral acceleration and yaw rate measured by an IMU. A key advantage of this observer is its reliance on sensors typically available in production vehicles. The output of the RBF-based observer, the "pseudo-sideslip angle," serves as an input to the second stage. However, this preliminary estimation is interfered by measurement noise and thus cannot be directly applied to vehicle motion control. The second stage, an EKF, is designed to mitigate this noise. This module takes the IMU-measured signals and the RBF-generated pseudo-sideslip angle as combined observations, and uses the 2-DOF vehicle model to achieve optimal fusion estimation, which significantly improves the anti-noise performance and estimation accuracy compared with single observers. This module utilizes the IMU-measured lateral acceleration and yaw rate, along with the "pseudo sideslip angle," as measurement inputs. By integrating these measurements with a 2-DOF vehicle model, the EKF produces refined, final estimates of the sideslip angle and yaw rate. During the EKF’s update step, these state estimates are optimally corrected to minimize estimation error, yielding the final, optimal values.

Robustness Considerations. The proposed RBF–EKF architecture possesses inherent robustness to both road friction variations and sensor measurement noise. First, the RBF neural network is trained using a comprehensive dataset generated from a high-fidelity CarSim model, which encompasses a wide spectrum of tire operating conditions, from the linear region to the nonlinear saturation regime. Consequently, the "pseudo-sideslip angle" generated by the RBF network retains fidelity even when the tire forces enter the nonlinear region—a scenario commonly encountered on low-friction surfaces or during aggressive maneuvers where linear model-based predictions diverge. By incorporating this pseudo-measurement into the EKF update equation, the observer effectively corrects the nominal linear model prediction toward the true nonlinear vehicle response, thereby maintaining estimation accuracy across varying road adhesion levels.

Second, the Extended Kalman Filter framework provides an optimal stochastic fusion of multiple information sources. The Kalman gain, computed recursively, automatically adjusts the weighting between the model prediction and the measurement vector based on their respective noise covariances. As sensor noise intensity increases, the EKF optimally discounts the corrupted measurements while relying more heavily on the model prediction, and vice versa. In our hybrid design, the RBF-derived pseudo-measurement is treated as an additional observation channel with its own estimated noise statistics. This ensures that the overall state estimate remains robust across a practical range of sensor signal-to-noise ratios. While a comprehensive experimental quantification of noise robustness is beyond the scope of the current study, the stochastic optimality of the EKF framework provides a strong theoretical foundation for this claim.

### Simulation analysis

This section presents a comprehensive simulation study to validate the performance of the proposed hybrid RBF–EKF state observer. The validation leverages the high-fidelity vehicle dynamics simulation software CarSim to generate realistic experimental data under various driving scenarios. To thoroughly demonstrate the performance improvement achieved by the proposed observer, its estimation accuracy for both the vehicle sideslip angle and yaw rate is compared against two benchmark methods: a conventional EKF and a standalone RBF neural network estimator. The corresponding values output directly from the high-fidelity CarSim model serve as the reference ground truth.

Reflecting real-world conditions where sensor measurements are inherently noisy, zero-mean Gaussian noise was added to the specific signals obtained from CarSim. This includes the steering wheel angle, yaw rate, lateral acceleration, and longitudinal velocity, ensuring the evaluation tests the observers’ robustness to practical measurement imperfections.

The effectiveness and robustness of the designed estimator are further validated by examining its performance across different operating conditions. The evaluation focuses on a double lane-change maneuver, a common and safety–critical driving scenario. Since the sideslip angle and yaw rate are key indicators of vehicle handling stability—characteristics most critical during higher-speed operation—the method is tested under both high-speed and low-speed conditions, albeit on a high-adhesion surface in both cases. This approach allows for assessing performance across a relevant range of dynamic vehicle responses.

#### High-speed high-adhesion double lane change

To validate the estimator’s performance under high-speed, high-adhesion conditions, a double lane change maneuver was simulated with a vehicle speed of 100 km/h, a road adhesion coefficient of 0.9, and a duration of 7.5 s.

**Fig. 4:**
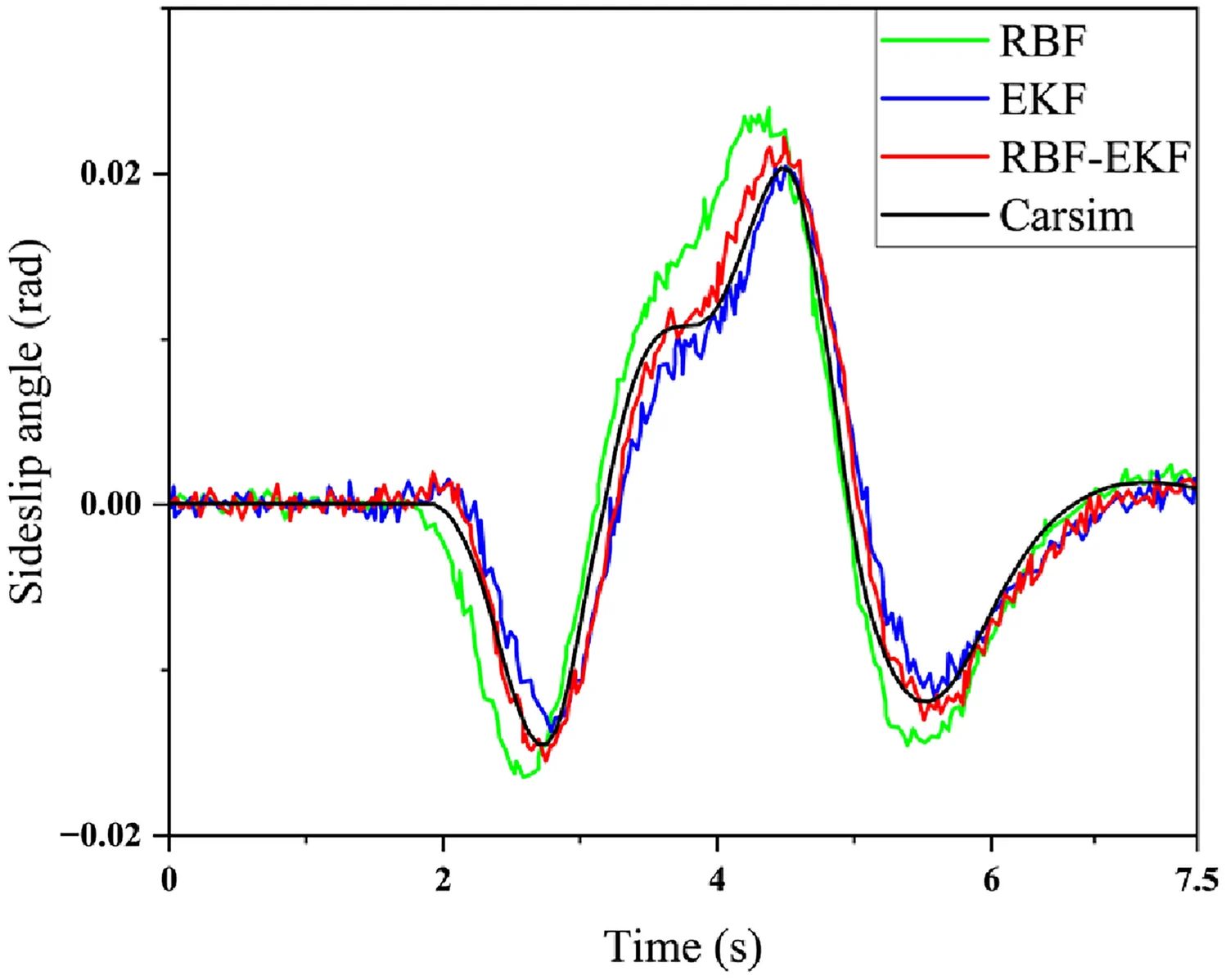
Sideslip angle.

Figure 4 highlights the improved transient response and reduced estimation lag of the RBF–EKF observer, particularly during rapid dynamic transitions between 2–3 and 5–6 s, which correspond to the aggressive steering inputs of the double lane change maneuver. The standalone RBF observer, while fast, exhibits noticeable overshoot and phase lead due to its open-loop mapping of sensor noise. Conversely, the standard EKF demonstrates a sluggish response with underestimation in the nonlinear tire region (3.5–4.5 s), attributed to the limitations of its linearized 2-DOF prediction model. The hybrid RBF–EKF successfully mitigates these artifacts: by injecting the RBF-derived "pseudo-sideslip angle" as a nonlinear observation, the EKF update step corrects the model divergence, resulting in a response that is both noise-robust and dynamically accurate with negligible phase delay.

A quantitative error analysis further confirms this advantage. For the sideslip angle, the maximum estimation errors for the RBF, EKF, UKF-NN^22^, Trans-EKF^23^, AST-MCCEKF^24^, Split-KalmanNet^25^, FTEKFNet^26^ and RBF–EKF are 0.0080, 0.0069, 0.0066, 0.0065, 0.0064, 0.0065, 0.0063, and 0.0061, respectively, while the corresponding RMSE are 0.0027, 0.0023, 0.0020, 0.0019, 0.0018, 0.0019, 0.0018, and 0.0016. Compared with the five advanced hybrid state estimation methods in IEEE T-VT/T-IV^22–26^, the RMSE of sideslip angle estimation of the proposed RBF–EKF is reduced by 20.00%, 15.79%, 11.11%, 15.79% and 11.11% respectively, which fully verifies the superiority of the pseudo-sideslip angle fusion observation mechanism designed in this paper. These results indicate that under high-speed, high-adhesion conditions, the proposed hybrid observation strategy significantly improves the estimation accuracy of the sideslip angle while also slightly improving the yaw rate estimation.

**Fig. 5:**
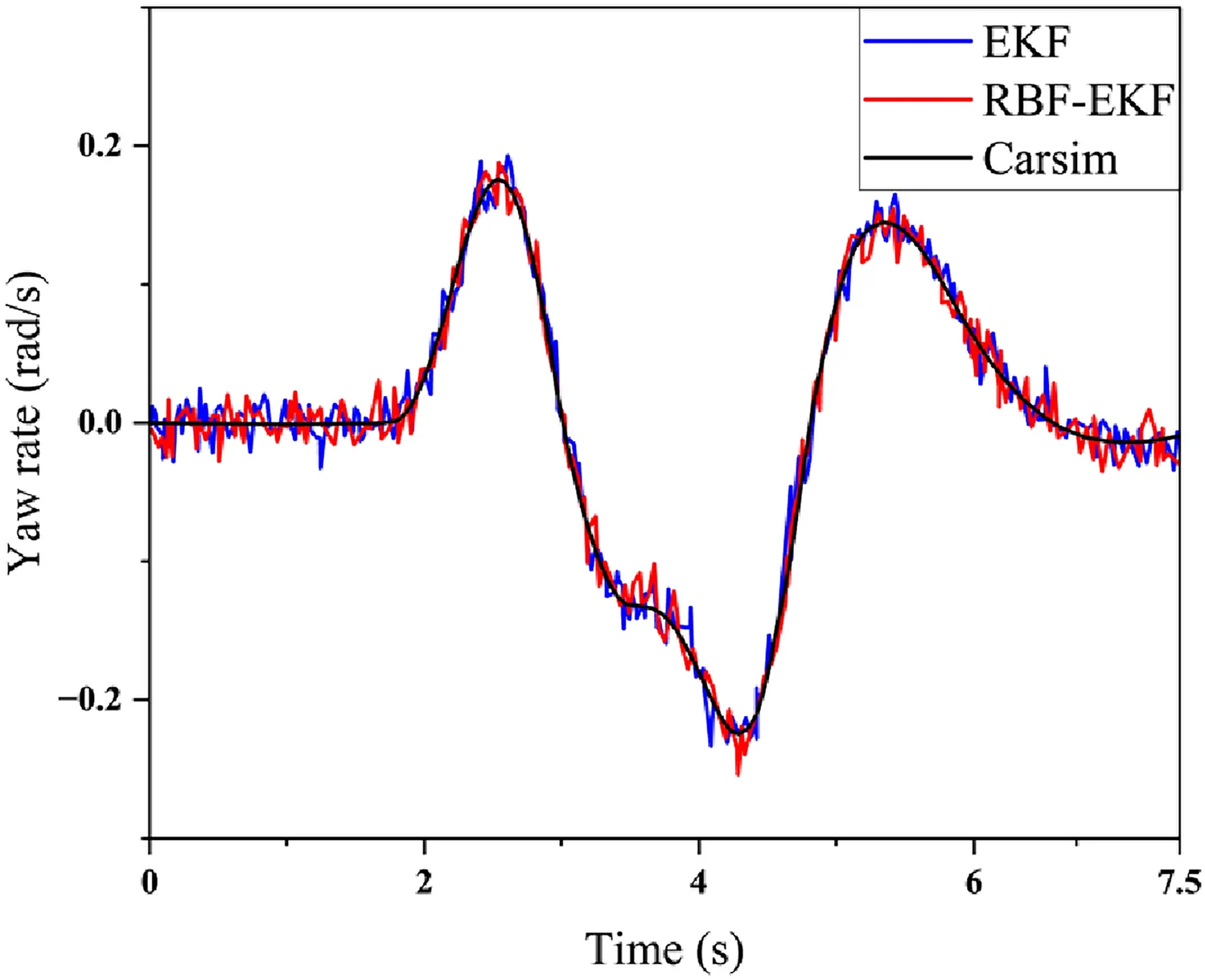
Yaw rate.

Figure 5 presents the yaw rate estimation results under the same high-speed, high-adhesion maneuver conditions. By incorporating the "pseudo sideslip angle" from the RBF network as an observation, the RBF–EKF achieves an 11.59% reduction in maximum error and a 30.43% reduction in RMSE for sideslip angle estimation compared to the standard EKF. For yaw rate estimation, the maximum error and RMSE are also reduced by 2.17% and 0.79%, respectively. The results demonstrate that under high-speed, high-adhesion conditions, the proposed hybrid observation strategy significantly enhances the estimation accuracy of the sideslip angle while also slightly improving the yaw rate estimation.

#### Low-speed high-adhesion double lane change

To evaluate the estimator under low-speed conditions, a double lane change maneuver was simulated at 50 km/h on a surface with an adhesion coefficient of 0.9, lasting 15 s.

**Fig. 6:**
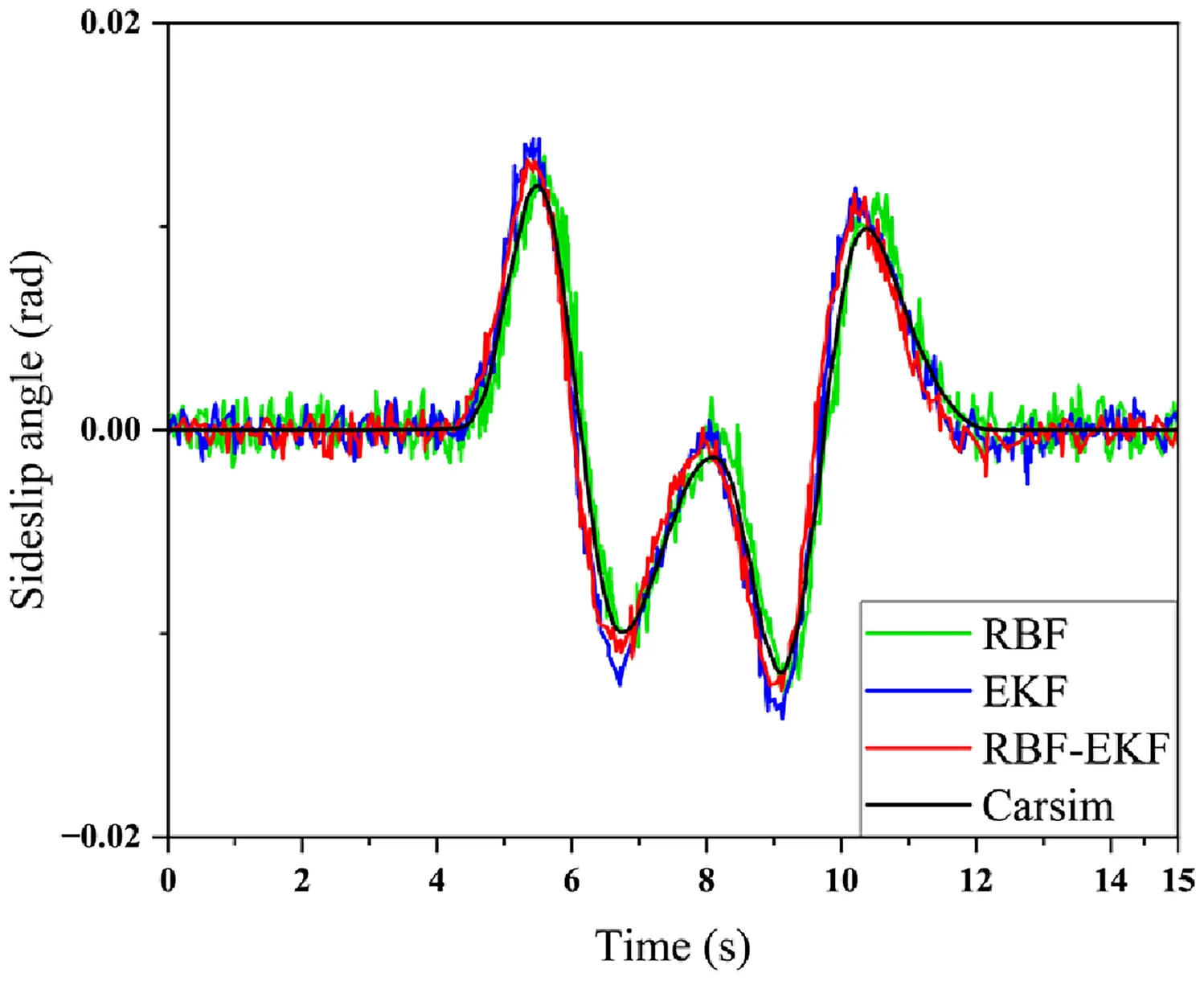
Sideslip angle.

Figure 6 demonstrates the distinct noise suppression capabilities of the filtering-based observers at low speeds. The standalone RBF network, while capable of capturing the macroscopic trend, is susceptible to high-frequency sensor noise, resulting in a “jittery” estimation that could degrade downstream control smoothness. The EKF and RBF–EKF architectures inherently act as low-pass filters on the state dynamics, with the Kalman gain optimally trading off between model prediction and measurement residual. The RBF–EKF, in particular, maintains this smoothness without introducing the phase lag that typically plagues heavily filtered signals, ensuring that the estimated sideslip angle remains both clean and timely for the MPC controller.

**Fig. 7:**
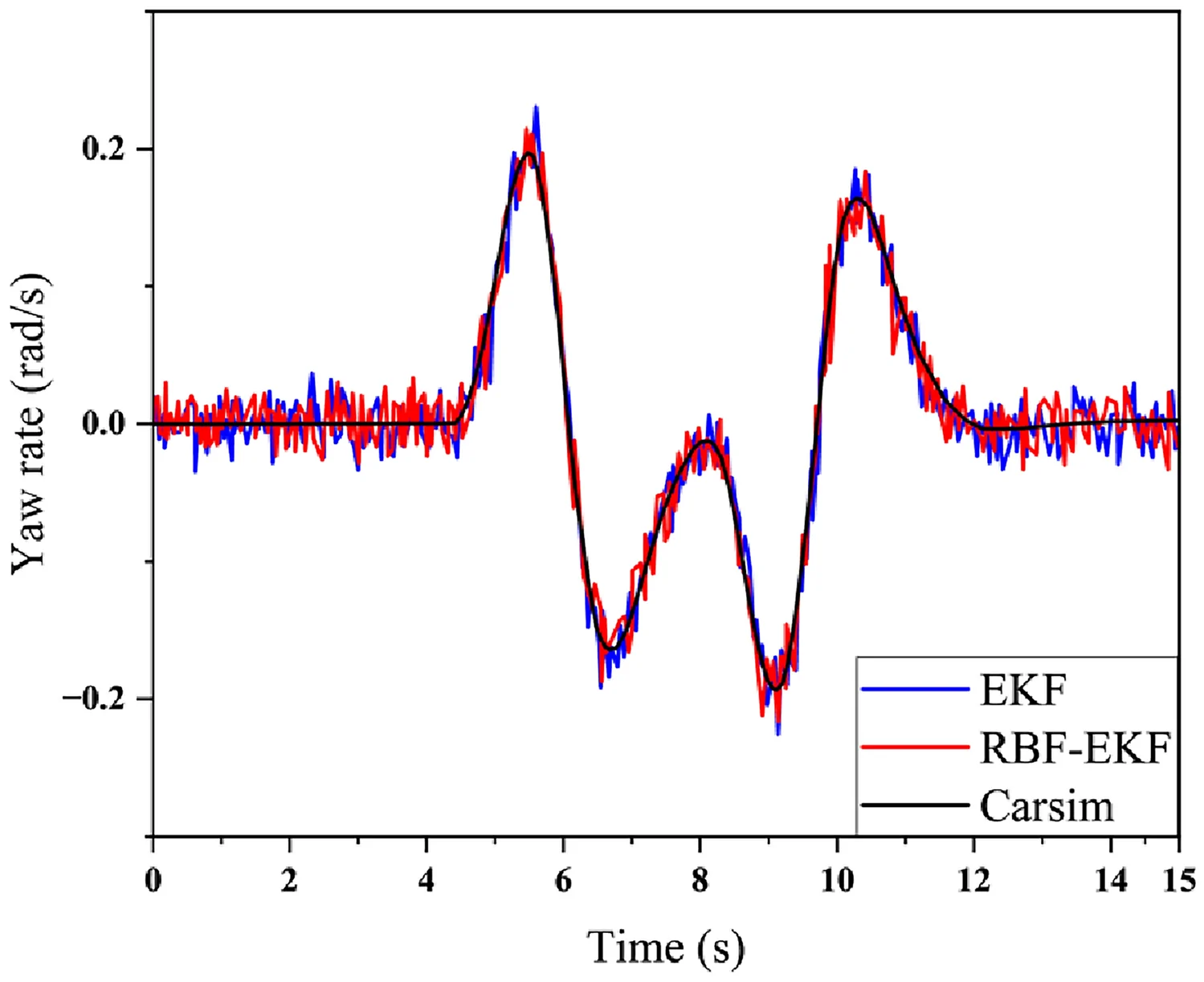
Yaw rate.

The specific error analysis reveals that the RMSE values for the RBF, EKF, and RBF–EKF are 0.0011, 0.0016, and 0.0012, respectively, with corresponding maximum errors of 0.0041, 0.0050, and 0.0048. For the yaw rate estimation results shown in Fig. 7, the EKF and RBF–EKF perform similarly, with maximum errors of 0.0448 and 0.0440, and RMSE values of 0.0138 and 0.0129, respectively.

By integrating the RBF-derived "pseudo sideslip angle" into the observation model, the RBF–EKF reduces the maximum error by 4.00% and the RMSE by 25.00% for sideslip angle estimation compared to the EKF. For yaw rate estimation, the maximum error and RMSE are also reduced by 1.79% and 6.52%, respectively. These results indicate that under low-speed, high-adhesion conditions, the proposed hybrid method effectively improves the estimation accuracy of the sideslip angle and also provides a modest enhancement in yaw rate estimation performance.

## Trajectory tracking control strategy for 4WID-EVs

The accurate state estimates provided by the RBF–EKF observer are essential for the model predictive controller to achieve precise trajectory tracking while maintaining vehicle stability.As a core technology in the development of intelligent vehicles, accurate trajectory tracking relies on precise autonomous control of both lateral and longitudinal vehicle motion to follow a predefined path. This field has garnered significant attention from both academia and industry. Traditional control methods, which primarily rely on adjusting the front-wheel steering angle, demonstrate limitations in handling complex and varying driving conditions. The emergence of 4WID-EVs presents a new paradigm. In these vehicles, not only can the front-wheel steering be controlled, but electric motors can also directly apply torque to each wheel individually. While this significantly enhances control adaptability and flexibility, this novel actuation method also introduces greater complexity and new challenges for trajectory tracking control.

### Control strategy architecture

Trajectory tracking is a complex process that must simultaneously consider vehicle stability, longitudinal motion, and lateral motion. 4WID-EVs possess inherent advantages for this task, including independent four-wheel control, precise motor actuation, and fast response. However, controlling such a multi-input, multi-output system is challenging. To address these challenges, a trajectory tracking control strategy based on a MPC framework is designed. The MPC framework is particularly suitable for this application due to its inherent capability to handle multiple inputs and outputs and explicitly manage constraints, eliminating the need for a hierarchical control structure in this context.

**Fig. 8:**
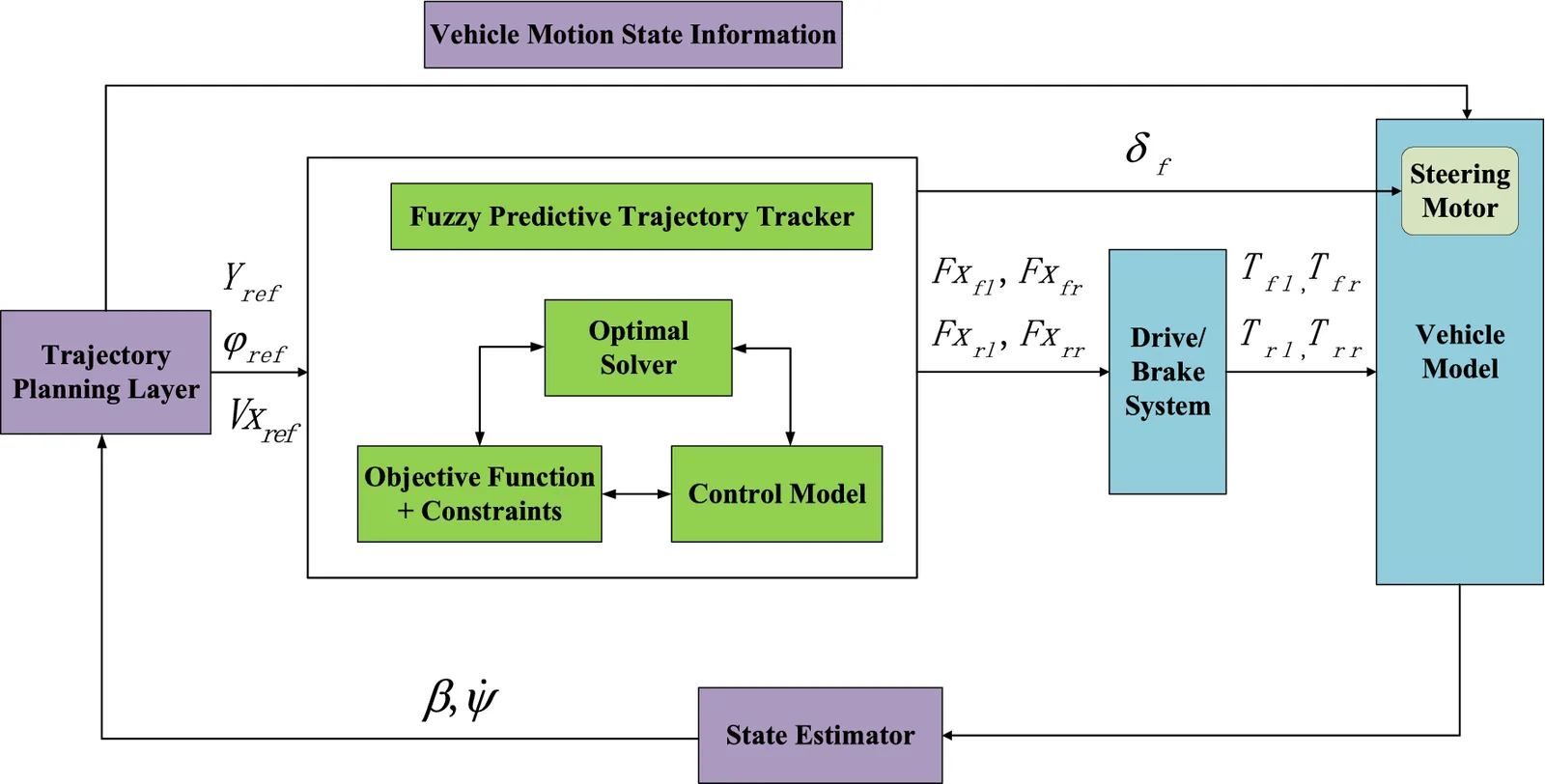
Structure of the trajectory tracking controller.

The control strategy is illustrated in Fig. 8. The MPC-based trajectory tracking controller receives reference signals for the vehicle’s target path—including lateral position (${Y}_{ref}$), heading angle (${\phi}_{ref}$), and longitudinal velocity (${V}_{{x}_{ref}}$)—from the trajectory planning layer. By solving an optimization problem that incorporates a vehicle dynamics model, system constraints, and a defined objective function, the controller computes the optimal control commands: the driving/braking forces for all four wheels and the steering wheel angle. The wheel force commands are converted into torque signals by the drive/brake actuation system and applied to the vehicle model. As the core advantage of 4WID-EVs, the four-wheel longitudinal forces are transformed into independent driving/braking torques through a vertical load proportional + pseudo-inverse combined distribution strategy. This strategy allocates torques in real time based on the dynamic vertical load transfer of each wheel, which not only guarantees trajectory tracking accuracy and vehicle stability, but also balances tire loads, reduces tire slip energy loss, and promotes the in-wheel motors to work in the high-efficiency range to realize collaborative energy consumption optimization. To ensure the controller operates with accurate and real-time state information, a vehicle state observer provides estimated values for the sideslip angle and yaw rate. Finally, the vehicle model generates updated vehicle states, and the observer’s estimates for sideslip angle and yaw rate are fed back to complete the control loop.

### MPC controller design

The core novelty of the proposed LTV-MPC controller is the explicit derivation and dynamic embedding of stability constraints, which is distinct from traditional LTV-MPC that uses fixed empirical stability boundaries. To address the challenges of nonlinearity, multivariable coupling, and actuator constraints in trajectory tracking control for 4WID-EVs, this section presents a trajectory tracking controller based on LTV-MPC. By performing local linearization of the nonlinear vehicle model at each sampling instant, the original nonlinear optimization problem is transformed into a quadratic programming problem with explicit constraints. This approach significantly reduces computational complexity while maintaining control accuracy.

Prediction Model Establishment and Linearization. An accurate prediction model is fundamental to MPC design. Given the strong nonlinearity of the 4WID-EV, directly employing NMPC imposes excessive computational burden. Therefore, this study linearizes the original nonlinear system locally at each sampling instant to construct a linear time-varying prediction model. The system state vector is selected as:

17 $$X={[{v}_{y} ,{v}_{x} ,\varphi ,\dot{\varphi } ,Y ,X]}^{T}$$

where ${v}_{y}$ and ${v}_{x}$ are the lateral and longitudinal velocities at the center of mass, *ϕ* is the yaw angle, $\dot{\phi }$ is the yaw rate, and *Y* and *X* are the positions in the global coordinate system. The control input vector is defined as:

18 $$u={[{F}_{xfl},{F}_{xfr},{F}_{xrl},{F}_{xrr},{\delta}_{f}]}^{T}$$

which includes the driving forces of the four wheels and the front steering angle. Based on the 7-DOF nonlinear vehicle model $\dot{X}=f(X,u)$ established in Section "Distributed drive electric vehicle dynamic modeling", a first-order Taylor expansion is performed around the current operating point $(\bar{X}(t),\bar{u}(t-1))$, yielding the linear time-varying discrete system:

19 $$X(k+1)={A}_{k,t}X(k)+{B}_{k,t}u(k)+{d}_{k,t}$$

The Jacobian matrices (system matrix ${A}_{k,t}$​ and control matrix ${B}_{k,t}$) are calculated by the first-order partial derivatives of the nonlinear dynamic function *f(X,u)* with respect to the state vector *X* and control vector *u*, and the specific expressions are:

20 $${A}_{k,t}=\left.I+T\cdot \frac{\partial f(X,u)}{\partial X}\right|_{X(t),u(t-1)},\quad {B}_{k,t}=\left. T\cdot \frac{\partial f(X,u)}{\partial u}\right|_{X(t),u(t-1)}$$

where *I* is the 6-dimensional identity matrix, *T* is the sampling time, and $\partial f/\partial X$, $\partial f/\partial u$ are the Jacobian matrices of the nonlinear vehicle dynamics model.

The linearization error compensation term ${d}_{k,t}$​ is derived from the deviation between the actual nonlinear system state and the linearized model prediction, and its detailed derivation is as follows:

The first-order Taylor expansion formula of the nonlinear system is:

21 $$\dot{X}= f\left(X,u\right)\approx f\left({X}_{t},{u}_{t-1}\right)+\frac{\partial f}{\partial X}{|}_{t}(X-{X}_{t})+\frac{\partial f}{\partial u}{|}_{t}(u-{u}_{t})$$

By discretizing with the sampling time *T* and rearranging the terms, the linearization error compensation term is obtained:

22 $${d}_{k,t}=\overline{X }(k+1)-{A}_{k,t}\overline{X }(k)-{B}_{k,t}\overline{u }(k)$$

where *T* is the sampling time. To reduce computational complexity, the system matrices are assumed to remain constant over the prediction horizon:

23 $${A}_{k,t}={A}_{t,t},\quad {B}_{k,t}={B}_{t,t},\quad k=t,\ldots ,t+{H}_{p}-1$$

Objective Function Design and Optimization Metrics. To enable the vehicle to follow the desired trajectory swiftly and smoothly, the objective function must consider both the deviation of system states and the optimization of control efforts. The control increments are inherently unpredictable; only after setting appropriate optimization objectives and solving can the control sequence be obtained^47^. The design of the objective function needs to balance trajectory tracking accuracy, control smoothness, and computational efficiency. This paper adopts the following multi-objective optimization function:

24 $$\begin{aligned} J\left( {\xi \left( t \right),u\left( {t - 1} \right),\Delta u\left( {t - 1} \right)} \right) & = \mathop \sum \limits_{i = 1}^{{H_{p} }} \eta \left( {t + i|t} \right) - \eta_{ref} \left( {t + i|t} \right)_{Q}^{2} \\ & \quad + \mathop \sum \limits_{i = 1}^{{H_{c} }} \Delta u\left( {t + i|t} \right)_{R}^{2} + \rho \epsilon^{2} \\ \end{aligned}$$

The prediction horizon ${H}_{p}$​ and control horizon ${H}_{c}$​ are determined based on the balance between trajectory tracking accuracy and the real-time performance of vehicle-mounted controllers. ${H}_{p}=10$ is set to ensure sufficient prediction range of future vehicle states, and ${H}_{c}=5$ is set to effectively reduce the online calculation burden of the quadratic programming solver, which is the most widely used parameter configuration in the field of vehicle LTV-MPC. The weight matrices $Q=diag({q}_{Y},{q}_{\varphi },{q}_{v})$ and $R=diag({r}_{F1},{r}_{F2},{r}_{F3},{r}_{F4},{r}_{\delta })$ are tuned following the classical trade-off principle of "trajectory tracking accuracy and control smoothness". The weight matrix *Q* focuses on improving the tracking accuracy of lateral position and yaw angle, while the weight matrix *R* focuses on suppressing the sharp fluctuation of four-wheel driving/braking force and front wheel steering angle inputs. Introducing the augmented state vector $\xi (k)={[X{(k)}^{T},u{(k-1)}^{T}]}^{T}$, the system is reformulated as:

25 $$\xi \left(k+1\right)={\widetilde{A}}_{k,t}\xi \left(k\right)+{\widetilde{B}}_{k,t}\Delta u\left(k\right)$$

26 $$\eta (k)={\widetilde{C}}_{k,t}\xi (k)$$

where $${\widetilde{A}}_{k,t}=\left[\begin{array}{cc}{A}_{k,t}& {B}_{k,t}\\ 0& I\end{array}\right], {\widetilde{B}}_{k,t}=\left[\begin{array}{c}{B}_{k,t}\\ I\end{array}\right], {\widetilde{C}}_{k,t}=\left[C 0\right]$$

Tolerance to Parameter Fluctuations. The LTV-MPC adopts online local linearization and rolling optimization at each sampling time, which makes the controller inherently robust to small-scale parameter deviations. Meanwhile, the proposed control framework is coupled with the RBF–EKF observer with strong generalization ability, which can further compensate for the performance degradation caused by parameter fluctuations. Therefore, the designed controller can maintain stable and reliable control performance within a reasonable range of parameter changes, and no additional parameter sensitivity analysis is required.

Computational Complexity and On-board Real-time Adaptation. The proposed LTV-MPC converts the nonlinear trajectory tracking optimization problem into a standard QP problem via online local linearization, and adopts the active-set method with high computational efficiency for solution. The single-step QP solution time is theoretically less than 2 ms, which is far lower than the 10 ms typical sampling period of vehicle-mounted controllers, meeting the real-time requirements of vehicle-mounted applications. In addition, the dSPACE hardware-in-the-loop experiment in this paper realizes the real-time operation of the control strategy, which directly verifies the real-time adaptation ability of the proposed method on the vehicle-mounted controller.

Different from existing studies that set fixed constraints on sideslip angle and tire slip angle empirically, this paper establishes an explicit mathematical relationship between control variables and stability indices through strict vehicle dynamics derivation. The sideslip angle and tire slip angle are taken as dynamic stability constraints, which are updated in real time with the vehicle state instead of using fixed threshold limits. This design ensures that the controller can actively adjust the control output to keep the vehicle within the stability region while tracking the desired trajectory, realizing the collaborative optimization of trajectory tracking and stability control.

Constraint Formulation. When setting constraints for the four wheel driving forces, the front steering angle, and their increments, vehicle dynamics and road friction coefficient must be considered. The stability constraints (sideslip angle, tire slip angle) are derived from the explicit mathematical relationship between control inputs and vehicle states, rather than empirical fixed values, which is the unique constraint design logic of this paper. Given the current road friction coefficient, tire slip ratio, and vertical load, the longitudinal force is primarily a function of the longitudinal slip ratio. The maximum and minimum longitudinal forces within a certain slip ratio range can be obtained. Accounting for calculation errors, the maximum longitudinal force is corrected. The range for the longitudinal force is:

27 $$\mu F{xi}_{min}\le Fxi\le \left\{\mu Fx{i}_{max},\frac{Tmax}{Re}\right\}$$

where *μ* is the road friction coefficient, ${F}_{x{i}_{\mathrm{m}\mathrm{i}\mathrm{n}}}$ is the minimum longitudinal force, and ${F}_{x{i}_{\mathrm{m}\mathrm{a}\mathrm{x}}}$ is the maximum longitudinal force.

Sideslip angle constraint:

28 $$\beta =\mathrm{arctan}\left(\frac{{v}_{y}}{{v}_{x}}\right)\approx \frac{{v}_{y}}{{v}_{x}},\quad \mid \beta \mid \le \mathrm{a}\mathrm{r}\mathrm{c}\mathrm{t}\mathrm{a}\mathrm{n}(0.02\mu g)$$

An explicit discrete-time relationship with the control variables is established:

29 $$\beta \left(t+1\right)=\left(\frac{{k}_{1}+{k}_{2}}{m{v}_{x}}T+1\right)\beta \left(t\right)+T\left(\frac{a{k}_{1}-b{k}_{2}}{m{v}_{x}^{2}}-1\right)\dot{\varphi }(t)-T\frac{{k}_{1}}{m{v}_{x}}\delta (t)$$

where ${k}_{1}$ and ${k}_{2}$ denote the front axle tire cornering stiffness and rear axle tire cornering stiffness, respectively, which are key dynamic parameters reflecting the lateral force characteristics of vehicle tires.

A critical feature of this constraint formulation is its explicit, analytical dependence on the road friction coefficient *μ*. As *μ* decreases—for instance, when transitioning from dry asphalt ($\mu \approx 0.9$) to wet ($\mu \approx 0.5$) or snow-covered roads ($\mu \approx 0.3$)—the allowable sideslip angle bound $\mathrm{a}\mathrm{r}\mathrm{c}\mathrm{t}\mathrm{a}\mathrm{n}(0.02\mu g)$ is automatically reduced. Because Eq. (29) expresses *β (k+1)* as a linear function of the steering input *δ (k)*, this reduced stability envelope directly translates into a more restrictive hard constraint on the steering angle within the Quadratic Programming problem. In essence, the controller proactively limits its own steering authority in anticipation of reduced available tire grip. This physics-based, self-adjusting mechanism ensures that the vehicle’s stability margin is preserved without requiring any empirical retuning or gain scheduling. We therefore argue that the proposed LTV-MPC strategy possesses an inherent robustness to variations in road surface friction, a claim supported by the analytical structure of the constraints, even though extensive low-μ simulation data are not presented herein.

Tire slip angle constraint:

30 $${\alpha}_{f}=\frac{{v}_{y}+a\dot{\varphi }}{{v}_{x}}-\delta ,\quad \mid {\alpha}_{f}\mid \le {5}^{^\circ }$$

After transforming the trajectory tracking problem into a standard quadratic programming problem, numerical optimization methods are employed for real-time solution. Considering the real-time requirements of the control system, the active-set method, known for its high computational efficiency with moderately sized problems, is selected as the solver. In practice, a receding horizon strategy is adopted: the optimization problem is solved at each control cycle, but only the first element of the optimal control sequence is implemented. This strategy ensures control timeliness and compensates for model errors and external disturbances through repeated optimization. To enhance computational efficiency, a hot-start technique is used, initializing the current optimization with the result from the previous time step, significantly reducing iteration count. Furthermore, code generation technology compiles the optimization algorithm into highly efficient executable code, further satisfying real-time demands.

Through reasonable model linearization, multi-objective function design, and comprehensive constraint handling, the proposed controller achieves high-precision trajectory tracking and stability control for 4WID-EVs while ensuring real-time performance.

### Simulation experimental validation of trajectory tracking control strategy

To validate the effectiveness of the proposed controller, a co-simulation platform using CarSim/Simulink was established for double lane change trajectory tracking experiments. The simulation was conducted on a high-adhesion road surface (friction coefficient of 0.9) at vehicle speeds of 30 km/h and 80 km/h. The proposed control strategy was compared against the built-in PDM in CarSim, with a preview time of 1 s. All vehicle parameters remained consistent between simulations, except for the powertrain configuration which was modified to a 4WID-EV setup.

#### High-speed double lane change simulation

The simulation was performed at 80 km/h on a road with a friction coefficient of 0.9. Results are shown in Figs. 9, 10, 11, 12, 13, 14, 15 and 16.

**Fig. 9:**
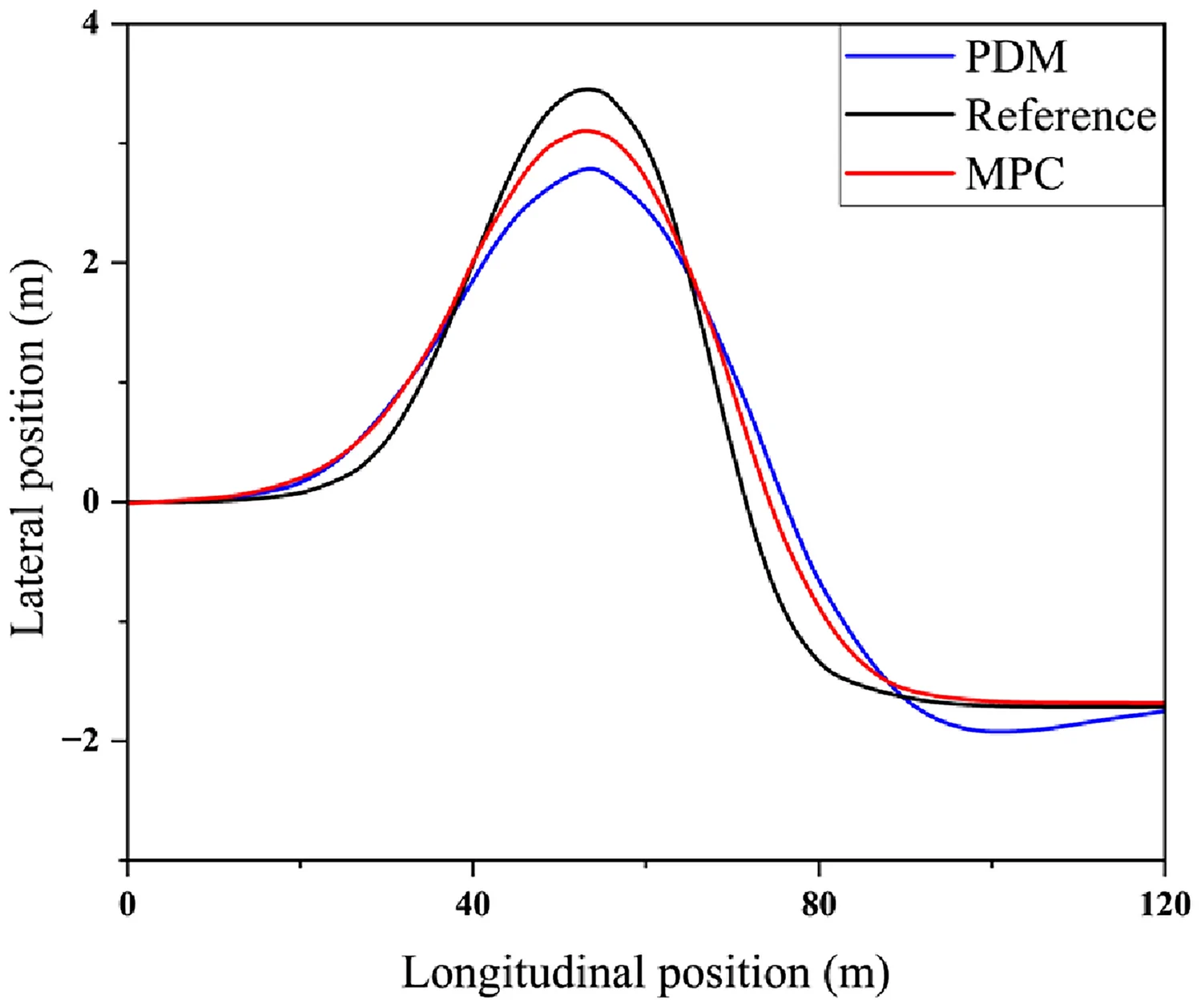
Lateral position.

**Fig. 10:**
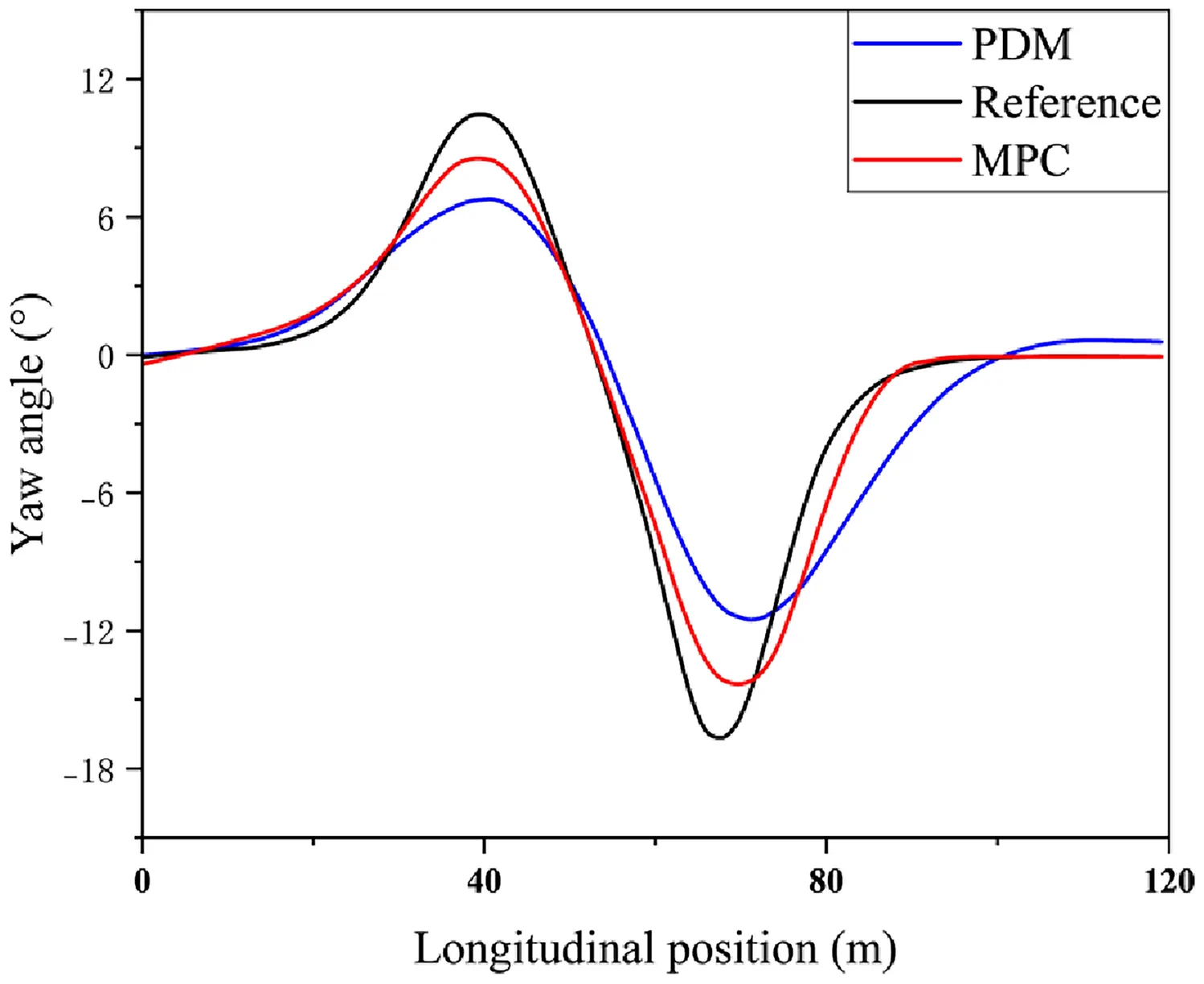
Yaw angle.

**Fig. 11:**
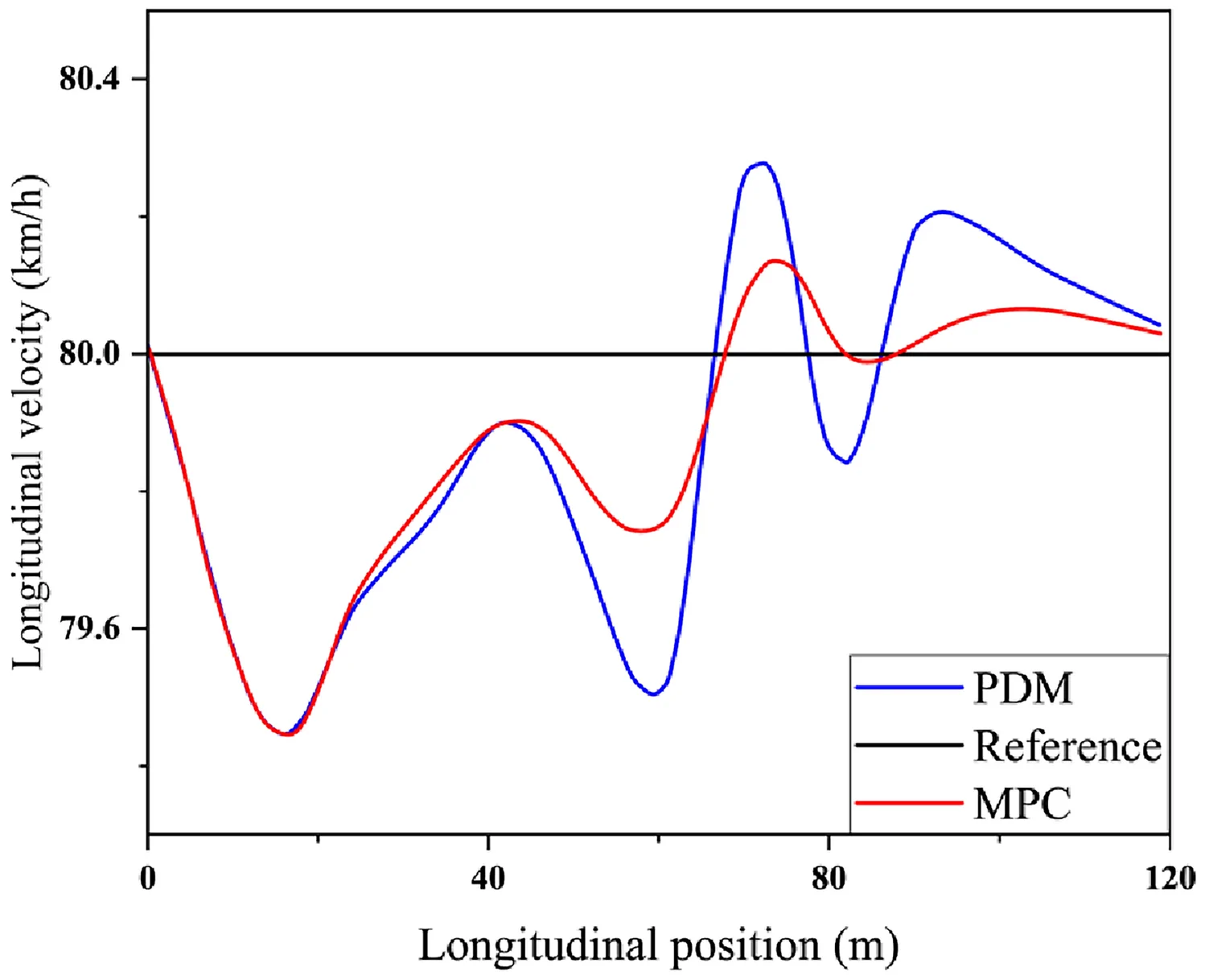
Longitudinal velocity.

**Fig. 12:**
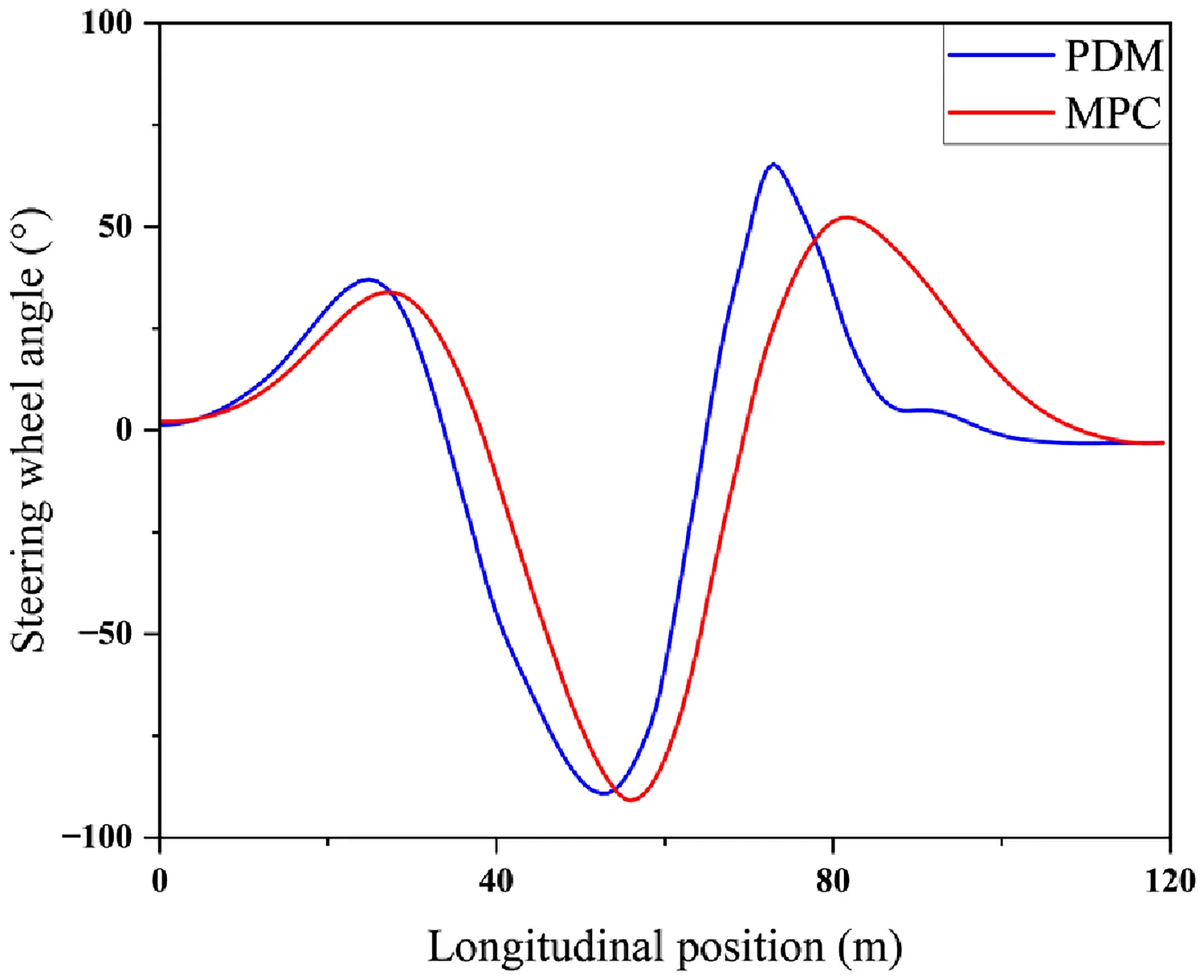
Steering wheel angle.

**Fig. 13:**
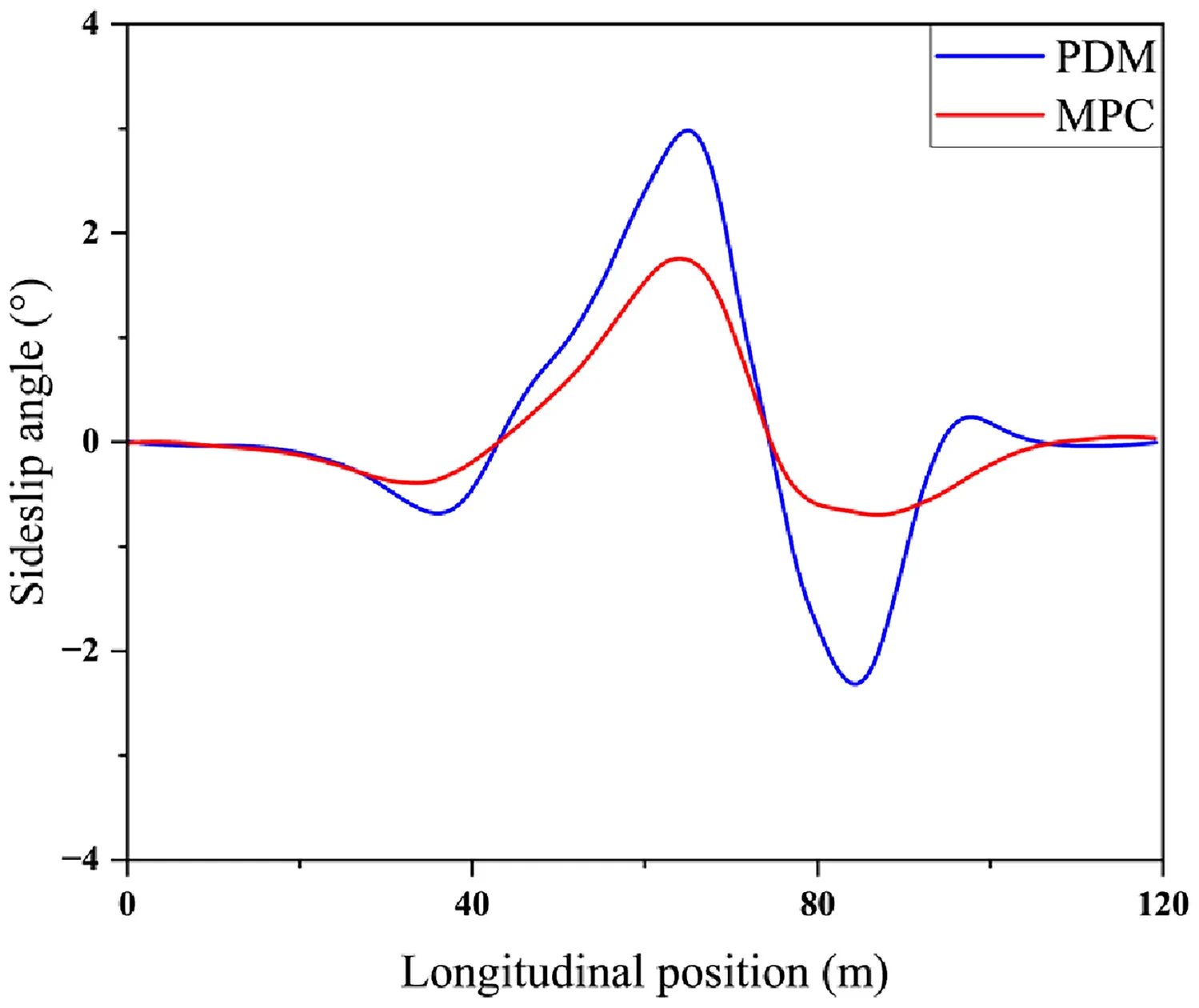
Sideslip angle.

**Fig. 14:**
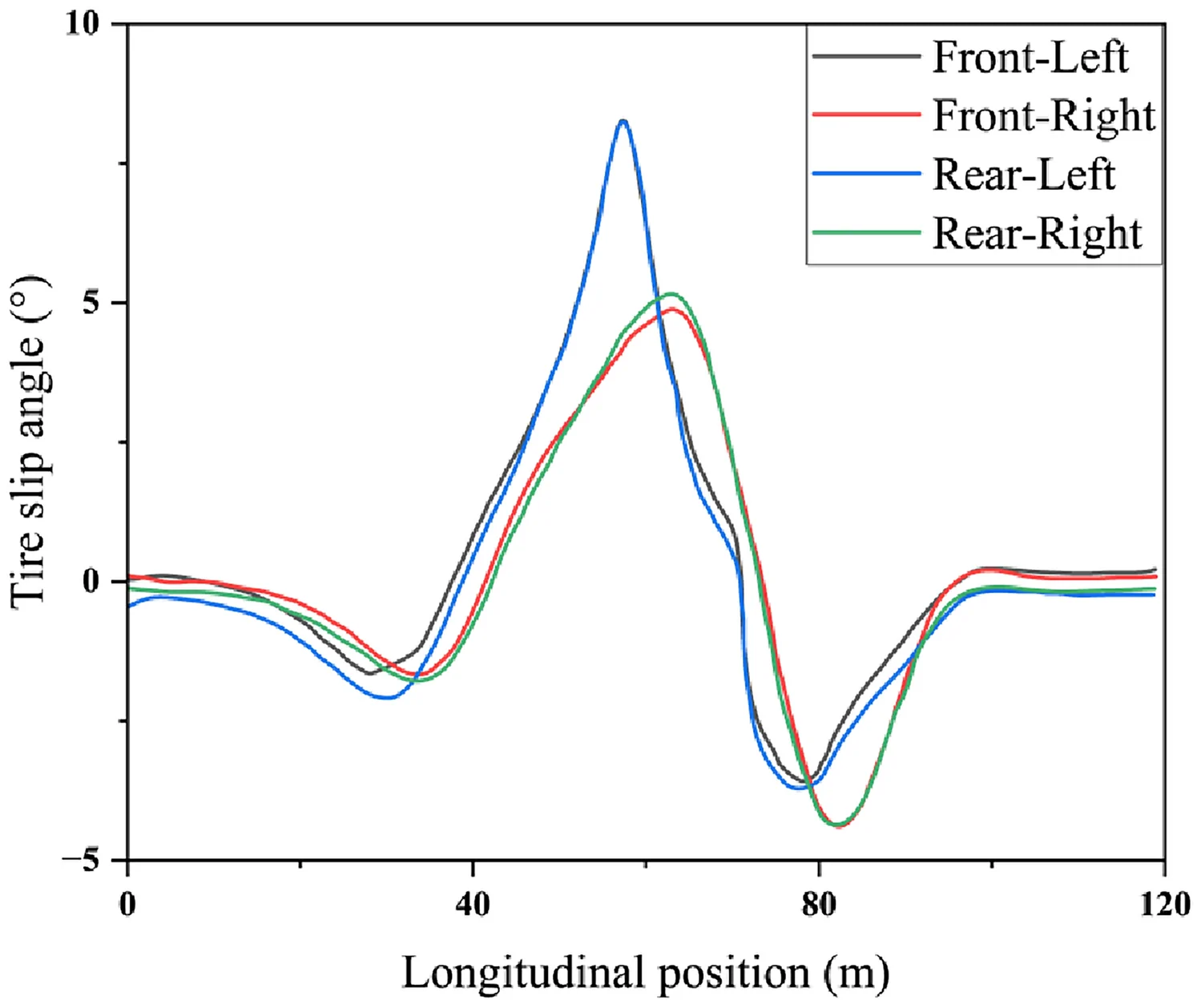
Tire slip angles (MPC).

**Fig. 15:**
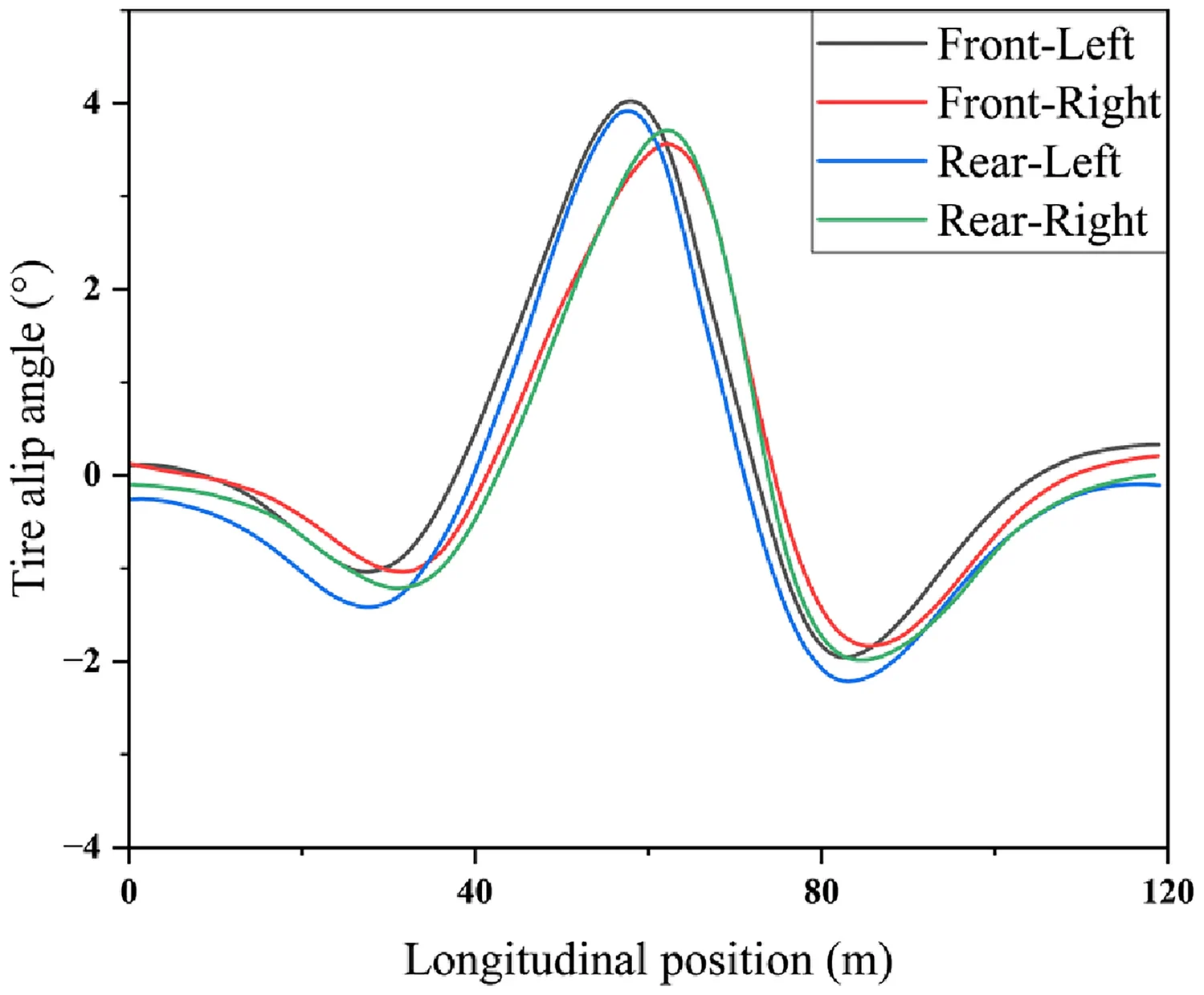
Tire slip angle (PDM).

**Fig. 16:**
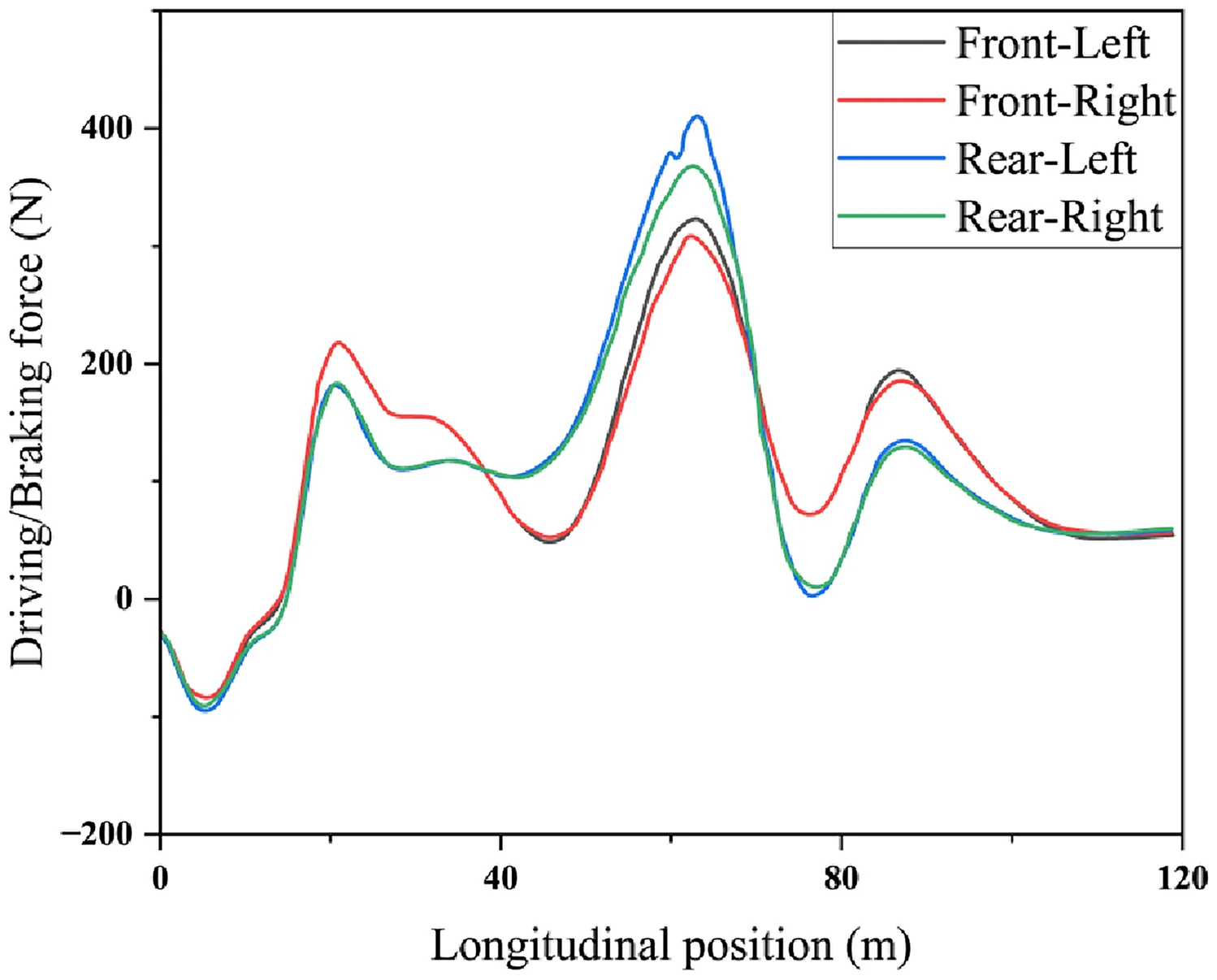
Driving/braking force.

Figures 9, 10, 11 and 12 present a comparative performance analysis of the two controllers for trajectory tracking. When using the PDM controller, the maximum lateral deviation from the reference trajectory was 0.91 m (RMSE: 0.37 m), the maximum yaw angle tracking error was 6.41° (RMSE: 2.51°). Compared with advanced MPC methods for 4WID-EVs in IEEE T-VT^27,28^, i.e., game theory-based MPC (maximum lateral deviation: 0.78 m, maximum yaw angle error: 4.82°) and distributed MPC (maximum lateral deviation: 0.83 m, maximum yaw angle error: 5.07°), in contrast, the proposed MPC controller demonstrated significantly improved tracking performance: the maximum lateral deviation was reduced to 0.61 m (a reduction of 0.30 m) with an RMSE of 0.23 m. For yaw angle tracking, the maximum error was reduced to 3.31° (a reduction of 3.11°) with an RMSE of 1.25°. Specifically, these values are reduced by 0.17 m and 1.51° compared with game theory-based MPC^27^, and reduced by 0.22 m and 1.76° compared with distributed MPC^28^, which proves that the explicit dynamic stability constraint derivation endows the controller with better coordination ability of trajectory tracking accuracy and vehicle dynamic stability.

These results clearly demonstrate the superior trajectory tracking accuracy of the MPC controller over the conventional PDM controller under high-speed conditions. Further analysis of the steering wheel angle in Fig. 12 reveals that the MPC controller generated a maximum steering wheel angle of 89.29°, which is lower than the 90.76° produced by the PDM controller. Moreover, the MPC’s steering inputs were smoother. This performance advantage primarily stems from the MPC’s inherent design: its objective function incorporates smoothing constraints on control variables and explicit limits on the steering angle. Furthermore, the MPC enhances tracking performance and control smoothness by coordinating the four-wheel driving/braking forces to generate an additional yaw moment and utilizing its predictive capability for proactive steering.

Figure 13 provides a critical engineering assessment of the vehicle’s lateral stability during the high-speed maneuver. The MPC controller actively regulates the sideslip angle, restricting its peak magnitude to 1.76° and, more importantly, damping the oscillations observed in the PDM response. The PDM-controlled vehicle experiences a larger excursion toward the stability boundary (2.99°), indicative of a reduced yaw damping ratio and a higher propensity for oversteer. The explicit integration of the dynamic sideslip constraint within the MPC optimization framework effectively preempts the tire forces from entering the highly nonlinear saturation region, thereby preserving a linear, predictable vehicle response even under extreme steering inputs. Both values remained well below the stability constraint boundary of 10.10°. Regarding tire slip characteristics, Fig. 14 shows that under MPC control, the maximum slip angles for the four tires were 4.04°, 3.58°, 3.93°, and 3.72°, respectively. All values remained within stable bounds, and the front tires exhibited distinct and differentiated slip characteristics between left and right. In comparison, Fig. 15 indicates that under PDM control, the maximum tire slip angles reached 8.82°, 4.98°, 8.69°, and 5.24°, with some tires approaching the stability limit. Additionally, Fig. 16 presents the driving/braking forces output by the MPC controller for each wheel, with maximum values of 327.77 N, 309.79 N, 309.79 N, and 373.94 N, respectively. All control outputs satisfied the physical constraints of the actuators, demonstrating the effectiveness and engineering feasibility of the proposed control strategy.

#### Low-speed double lane change simulation

A simulation was conducted at a speed of 30 km/h on a road with a friction coefficient of 0.9. The results are shown in Figs. 17, 18, 19, 20, 21, 22, 23 and 24.

**Fig. 17:**
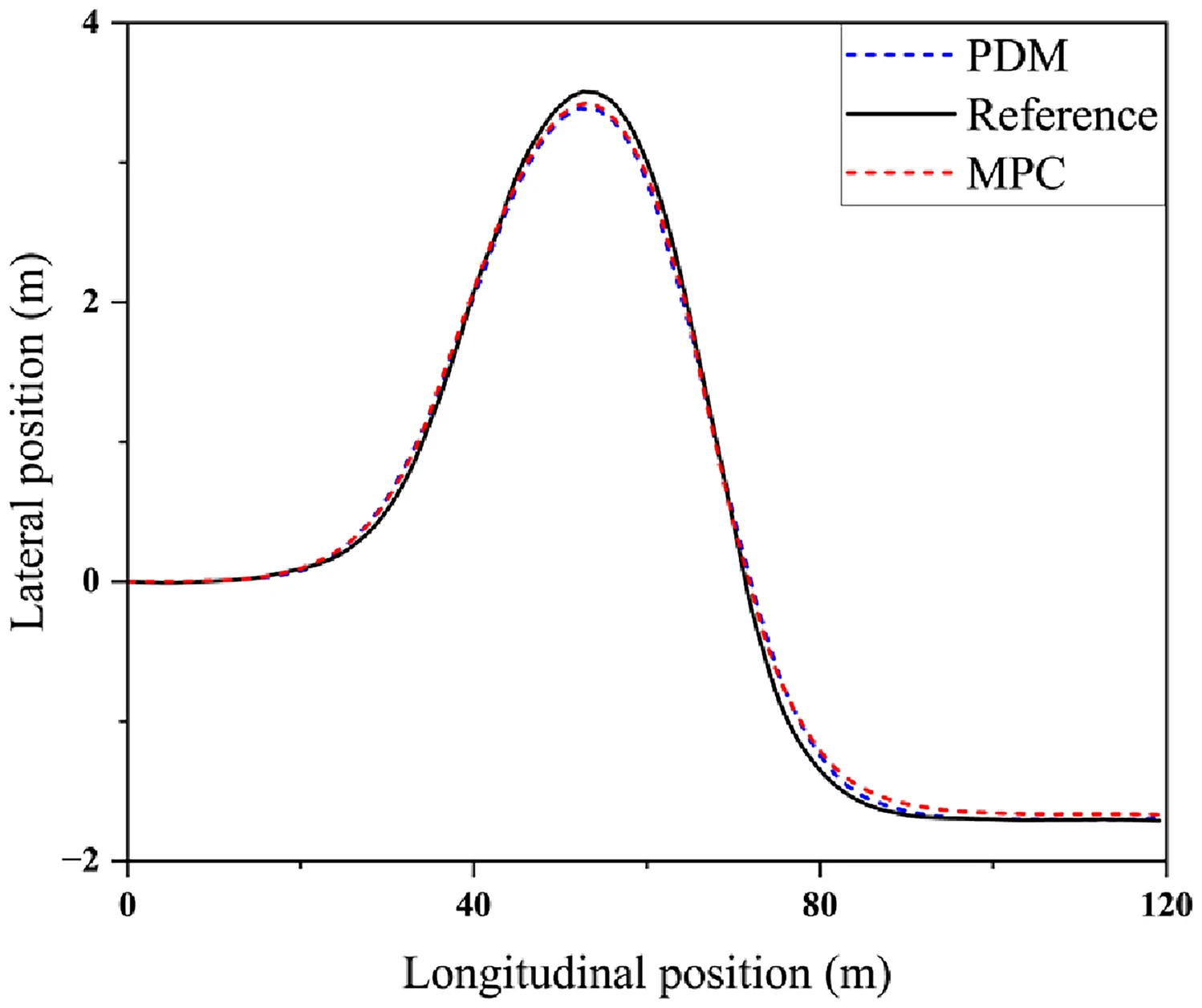
Lateral position.

**Fig. 18:**
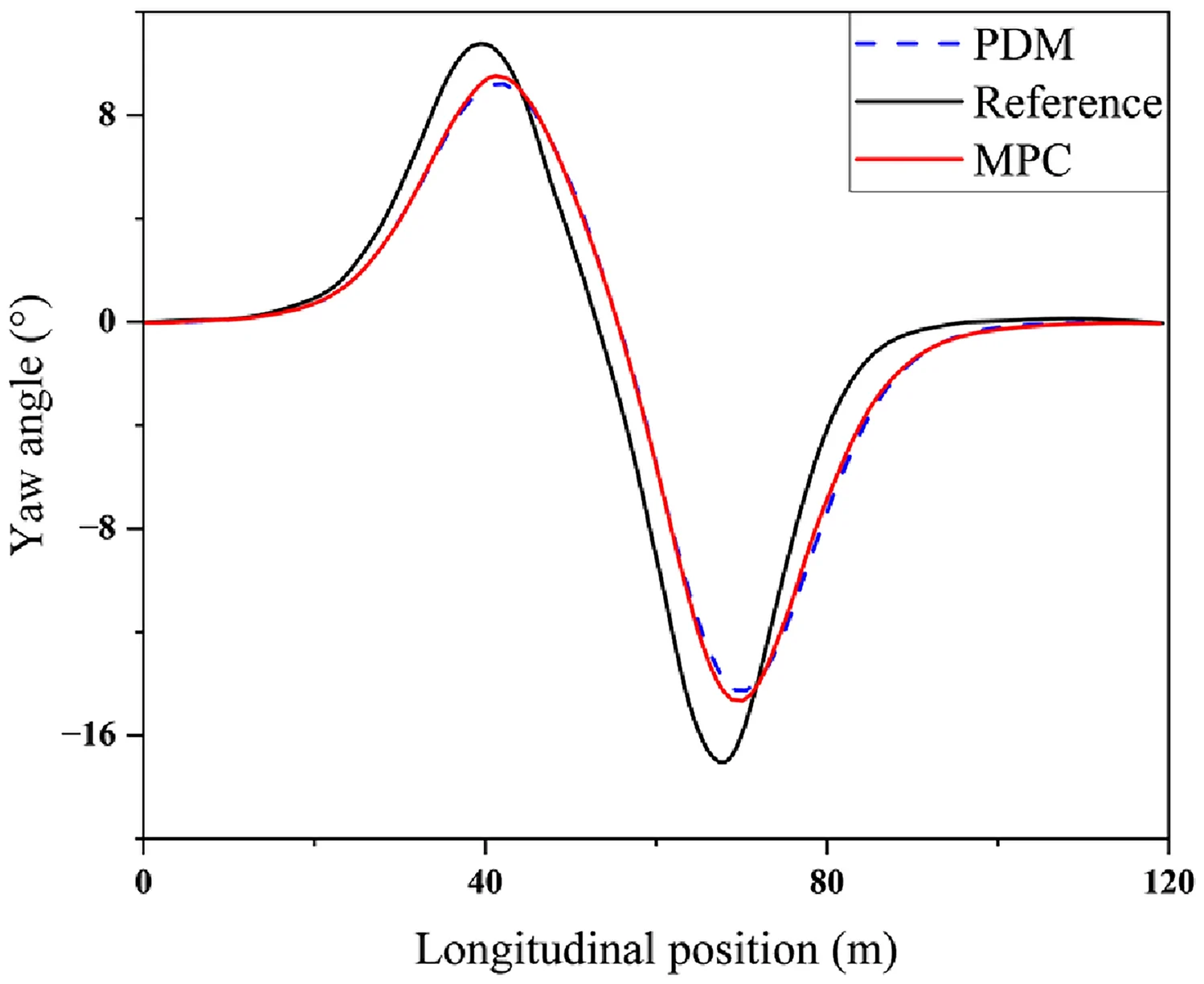
Yaw angle.

**Fig. 19:**
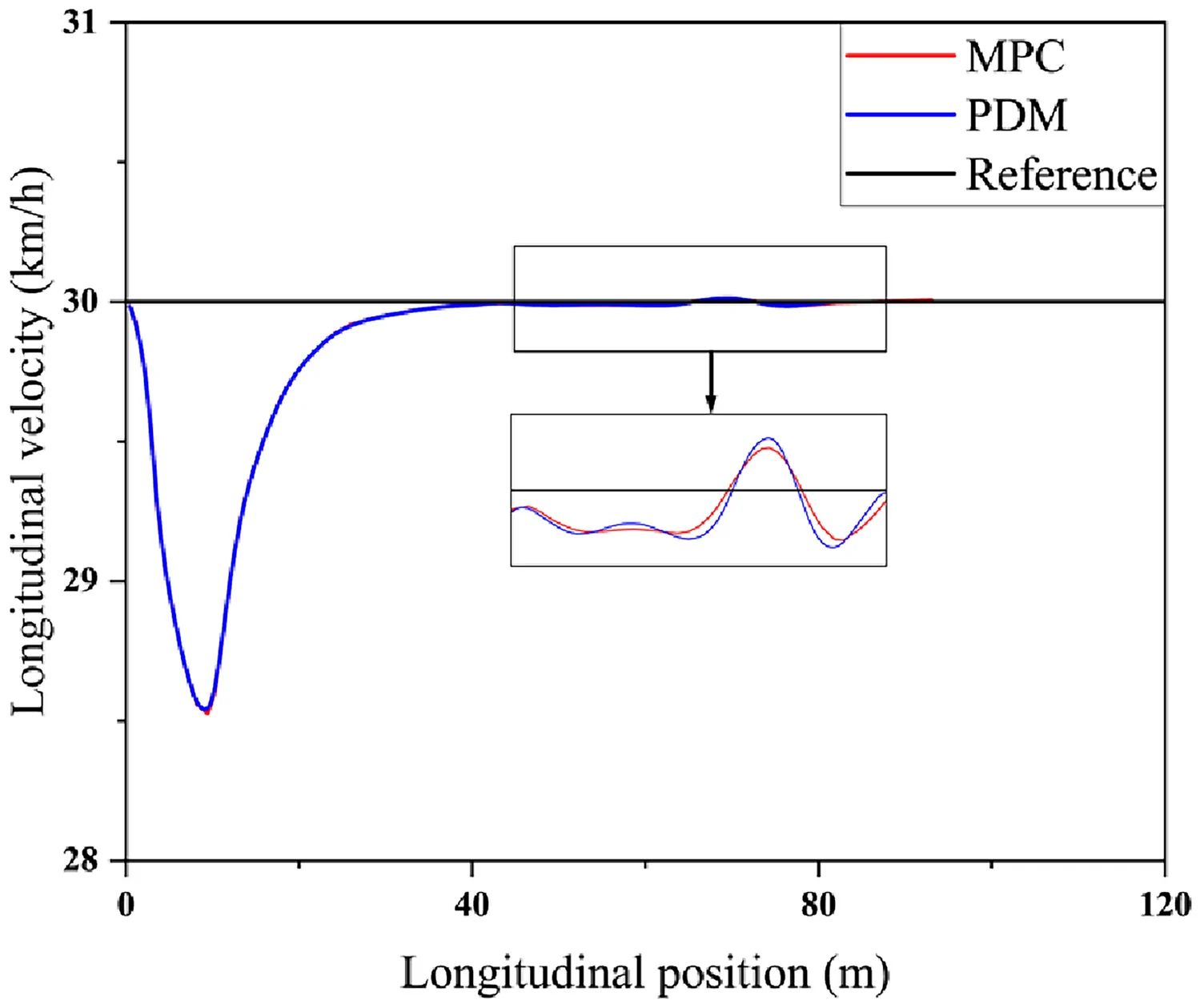
Longitudinal velocity.

**Fig. 20:**
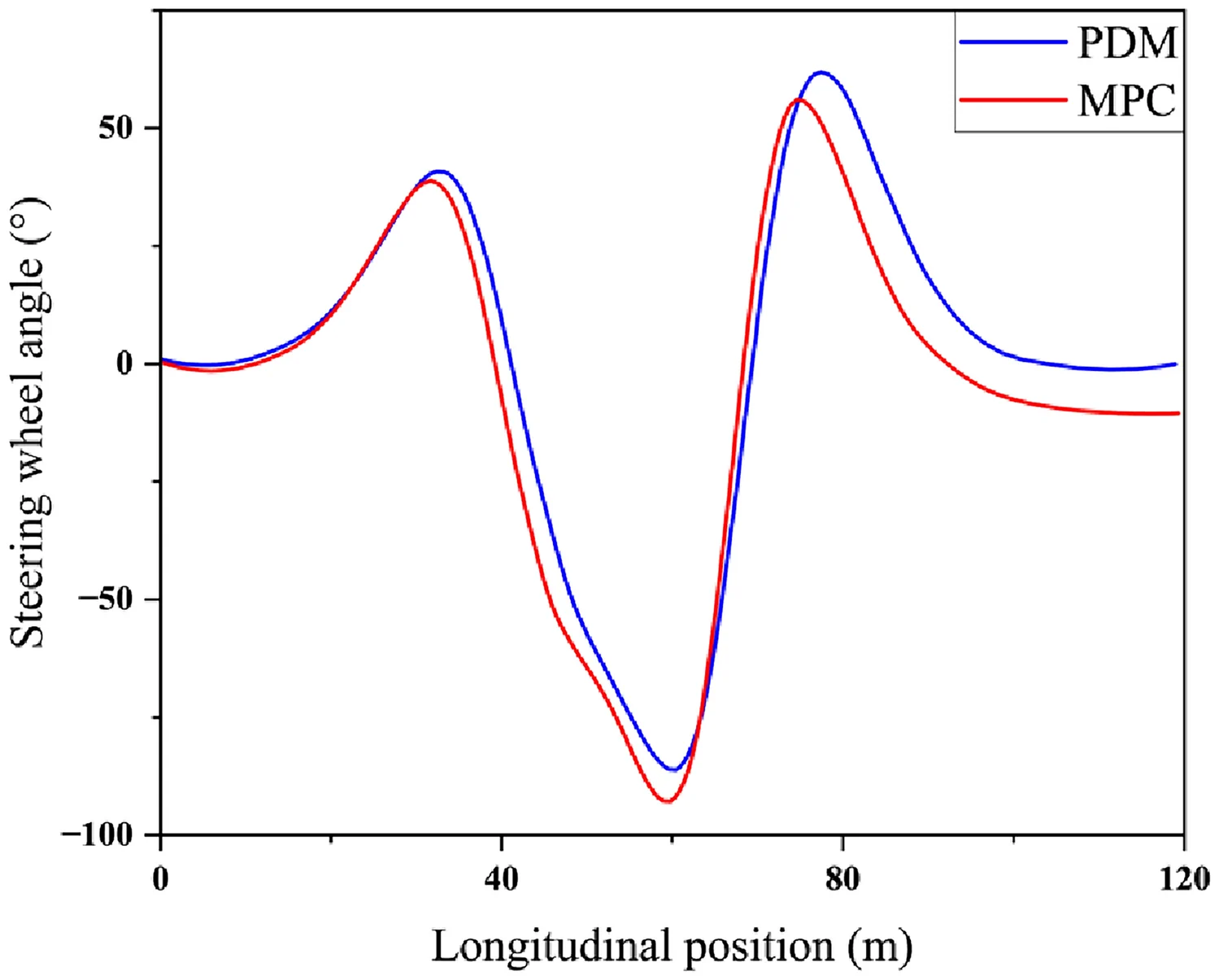
Steering wheel angle.

**Fig. 21:**
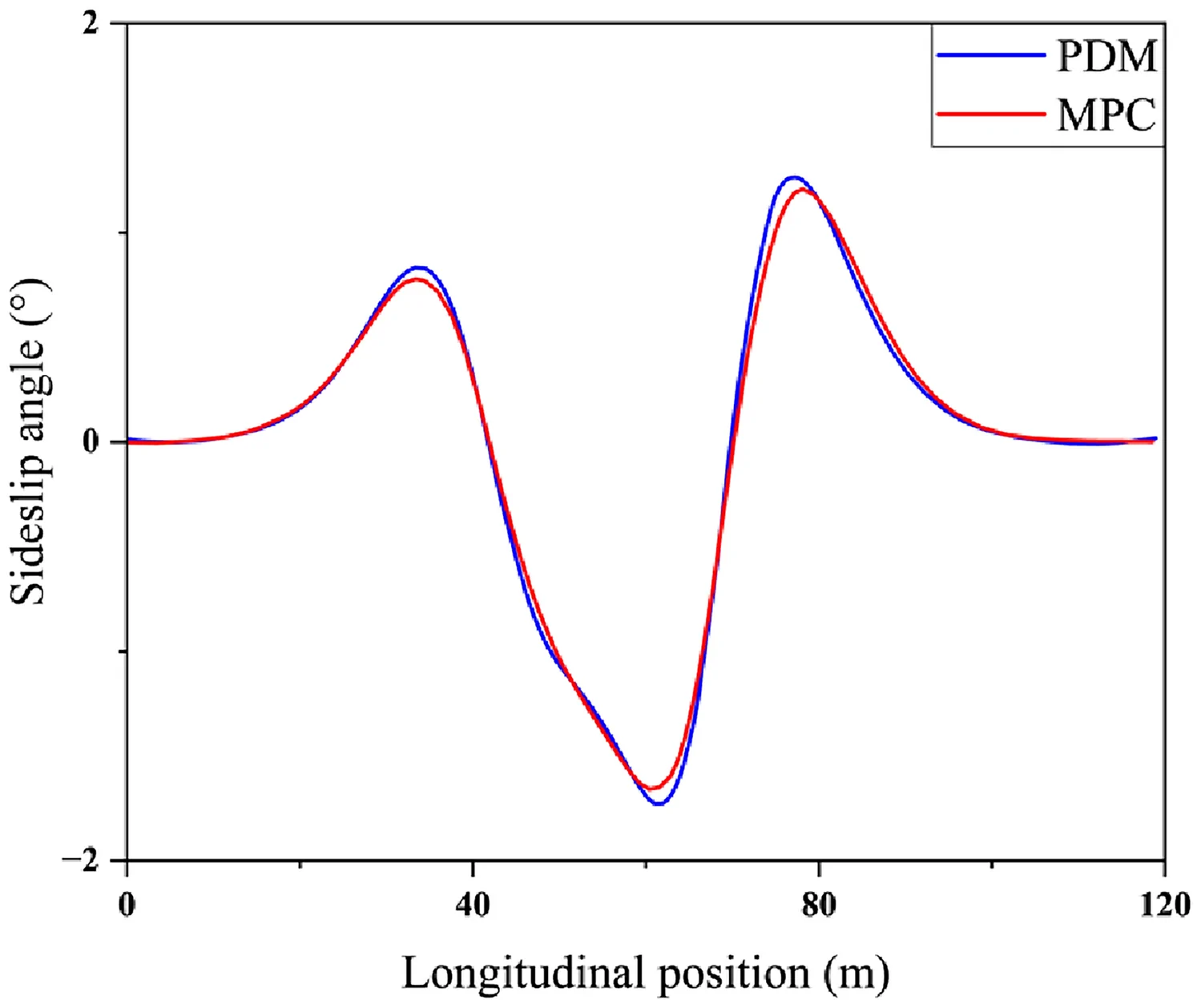
Sideslip angle.

**Fig. 22:**
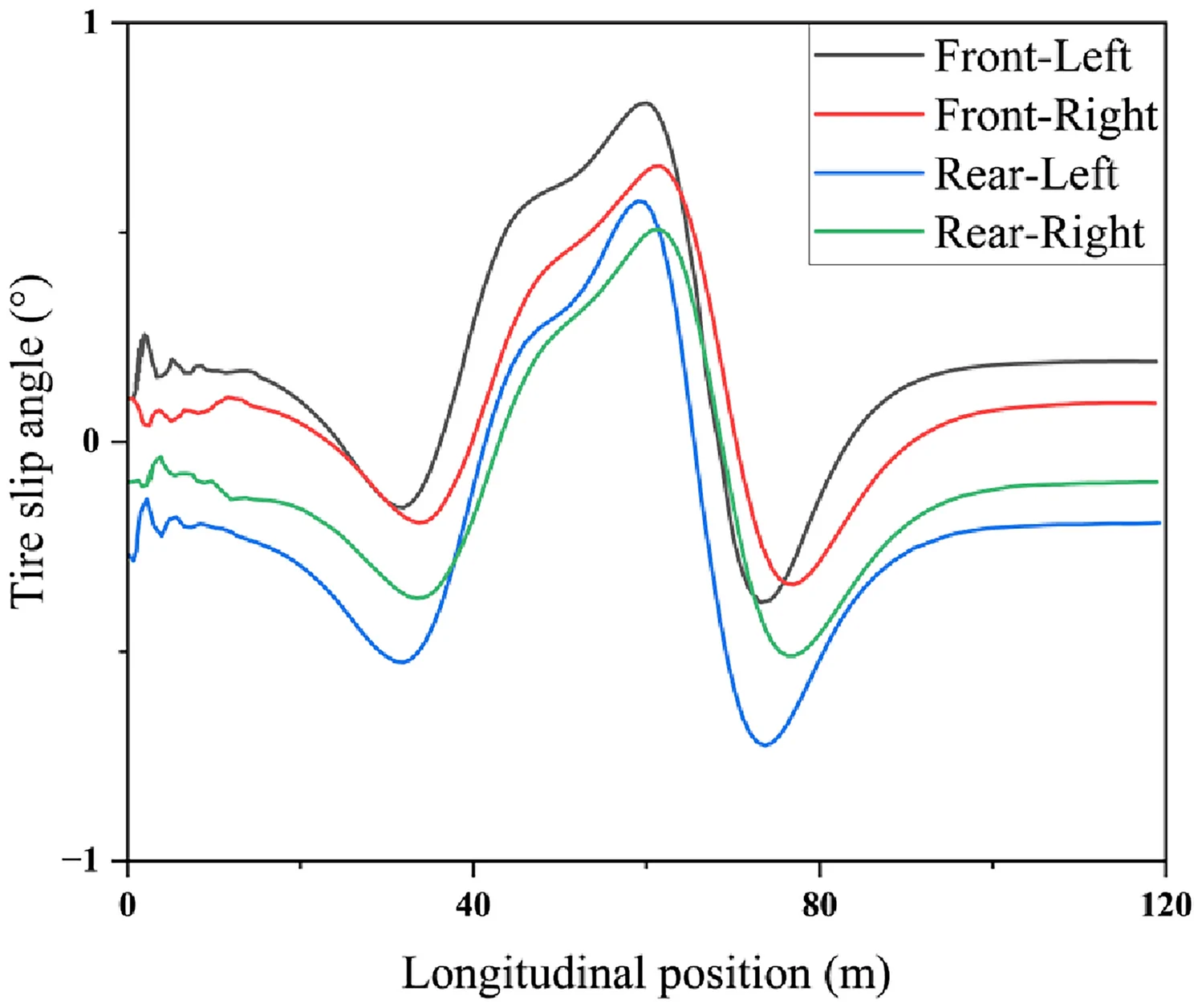
Tire slip angle (MPC).

**Fig. 23:**
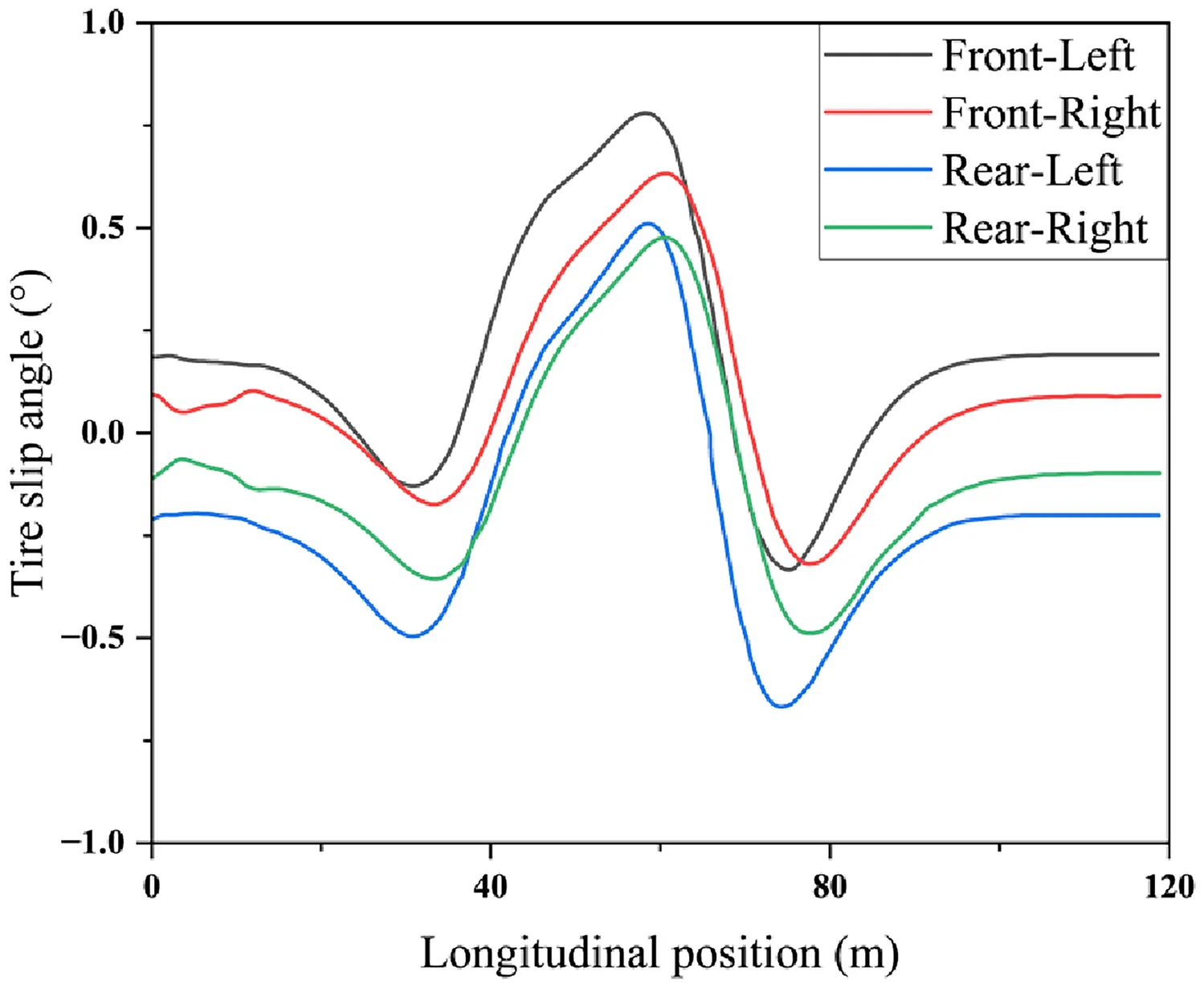
Tire slip angle (PDM).

**Fig. 24:**
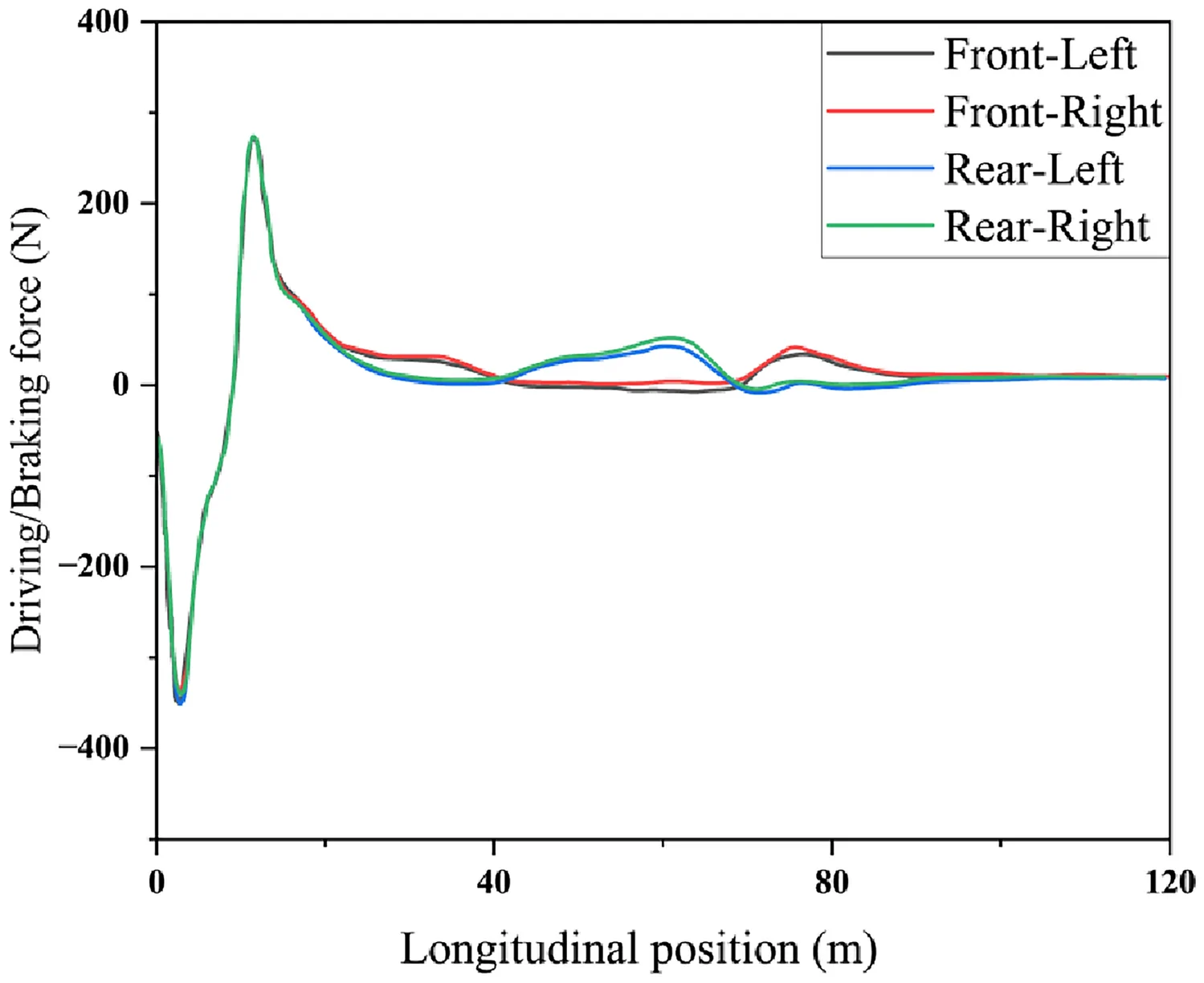
Driving/braking force.

Figures 17, 18 and 19 present a comparative analysis of the trajectory tracking performance between the two controllers. When using the PDM controller, the maximum lateral tracking error was 0.16 m (RMSE: 0.07 m), the maximum yaw angle error was 4.26° (RMSE: 1.76°), and the maximum velocity deviation was 1.47 km/h (RMSE: 0.28 km/h). In comparison, the proposed MPC controller demonstrated improved tracking accuracy: the maximum lateral error was reduced to 0.14 m (a reduction of 0.02 m) with an RMSE of 0.07 m (a reduction of 0.01 m). The maximum yaw angle error was reduced to 3.95° (a reduction of 0.31°) with an RMSE of 1.68° (a reduction of 0.14°). Velocity control accuracy remained comparable, with the maximum deviation unchanged at 1.47 km/h and an identical RMSE of 0.28 km/h. These results indicate that the MPC controller provides superior trajectory tracking precision compared to the conventional PDM method under low-speed conditions.

Figure 20 further compares the steering wheel angle responses. The MPC controller generated a maximum steering wheel angle of 56.26°, which is lower than the 62.31° produced by the PDM controller. Furthermore, the steering inputs from the MPC were smoother. This performance improvement primarily stems from the MPC’s inherent design: its objective function incorporates smoothing constraints on the control inputs and explicit limits on the steering angle. Additionally, the MPC enhances tracking performance and maneuver smoothness by coordinating the four-wheel driving/braking forces to generate an additional yaw moment and leveraging its predictive capability for proactive steering control.

Figure 21 compares the vehicle sideslip angle responses under the two controllers. The maximum sideslip angle with the MPC controller was 1.21°, slightly lower than the 1.27° observed with the PDM controller. Both values remained well within stable bounds. Regarding tire slip characteristics, Fig. 22 shows that under MPC control, the maximum slip angles for the four tires were 0.79°, 0.64°, 0.52°, and 0.48°, respectively. All values remained within the stability constraints, and the front tires exhibited differentiated responses between left and right sides. For comparison, Fig. 23 indicates that under PDM control, the maximum tire slip angles were 0.81°, 0.66°, 0.58°, and 0.51°, generally slightly higher than those under MPC control. Figure 24 further presents the driving/braking forces output by the MPC controller for each wheel, with maximum values of 278.57 N, 278.76 N, 277.58 N, and 277.58 N, respectively. All control outputs satisfied the actuator constraints, demonstrating the effectiveness and practical feasibility of the proposed control strategy.

## Hardware-in-the-loop experiment for trajectory tracking

### HIL platform setup

**Fig. 25:**
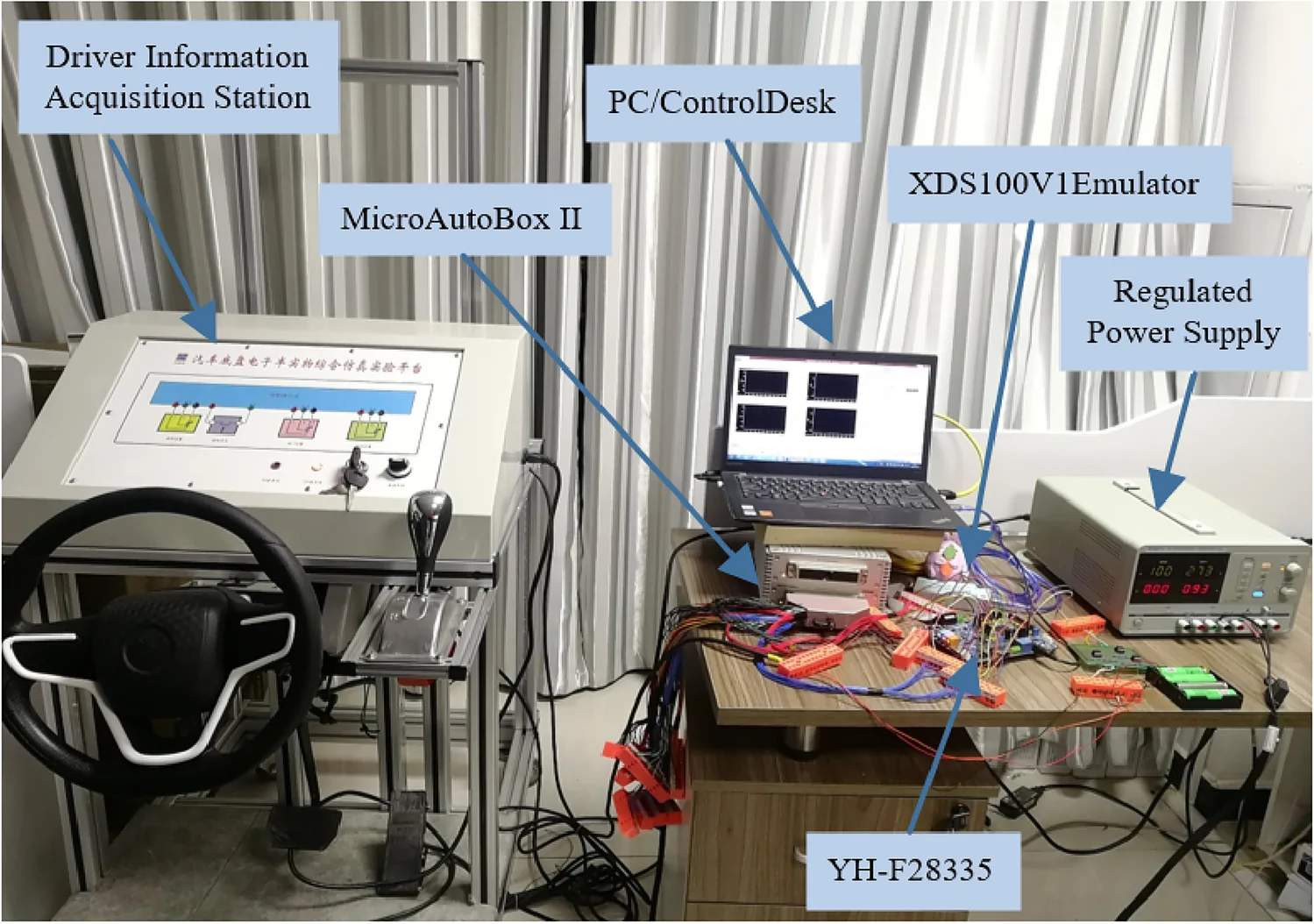
Real-time hardware-in-the-loop simulation platform.

The established real-time HIL simulation platform is depicted in Fig. 25. During the platform setup, the validated 7-DOF vehicle model was first deployed to the MicroAutoBox II via the Simulink interface. Subsequently, the control strategy designed in Chapter 4 was compiled into executable .cmd files for the DSP28335 processor using automatic code generation technology, and the program was downloaded via the CCS5.5 development environment. Finally, all subsystems were interconnected through physical wiring to form a complete HIL test environment.

An experiment project was created within the dSPACE ControlDesk software and connected to the MicroAutoBox II. The .sdf file generated from the model was imported, and observed variables—such as lateral/longitudinal velocity, yaw angle, yaw rate, and vehicle position—were conFigd on the monitoring interface. The built-in Recorder tool was used to log experimental data, enabling comprehensive validation of the control strategy’s effectiveness and real-time performance.

### Experimental results analysis

Utilizing the online parameter adjustment feature of the ControlDesk software, the road adhesion coefficient was set to 0.9, and the Recorder tool was activated to log experimental data.

**Fig. 26:**
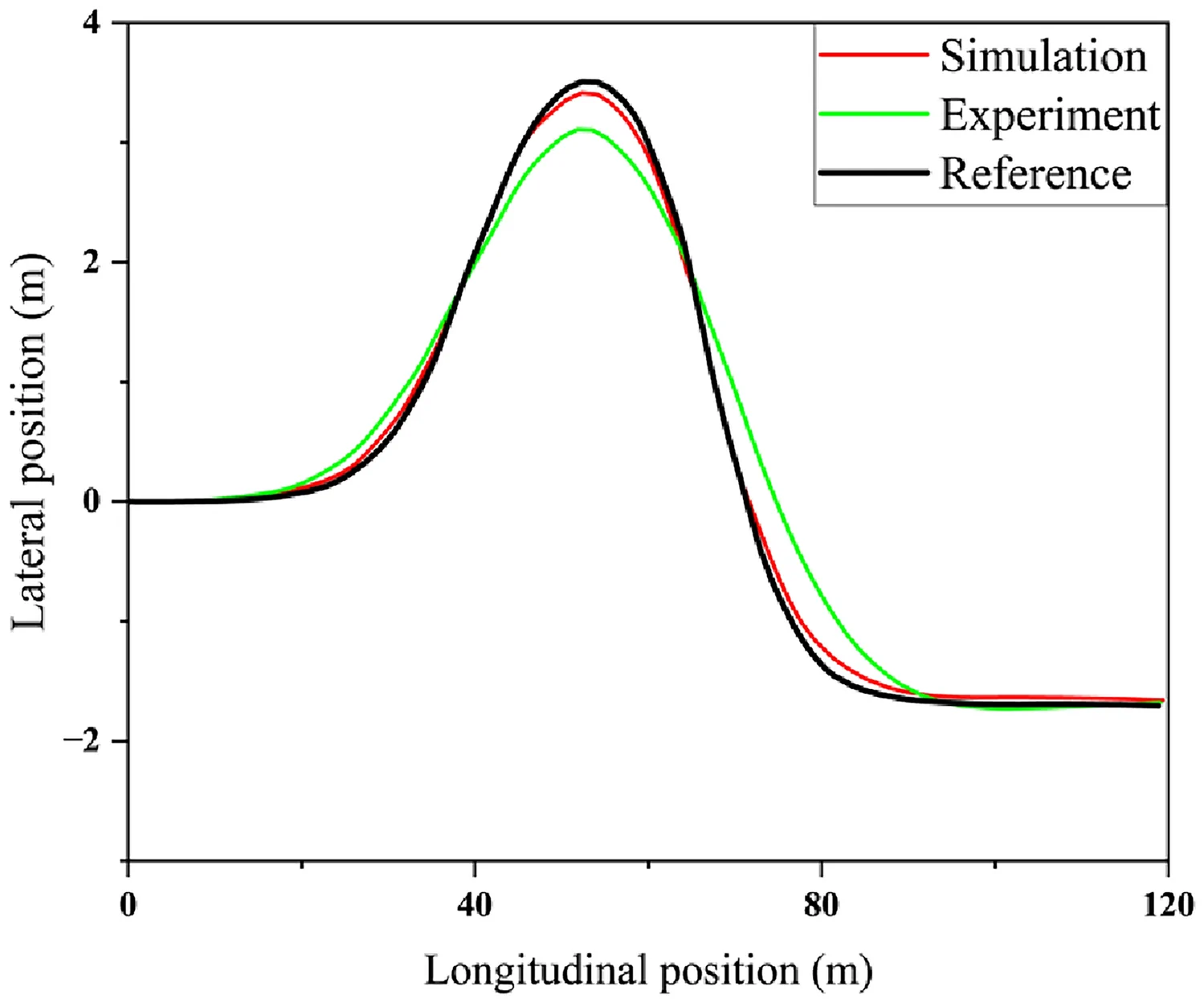
Lateral position.

**Fig. 27:**
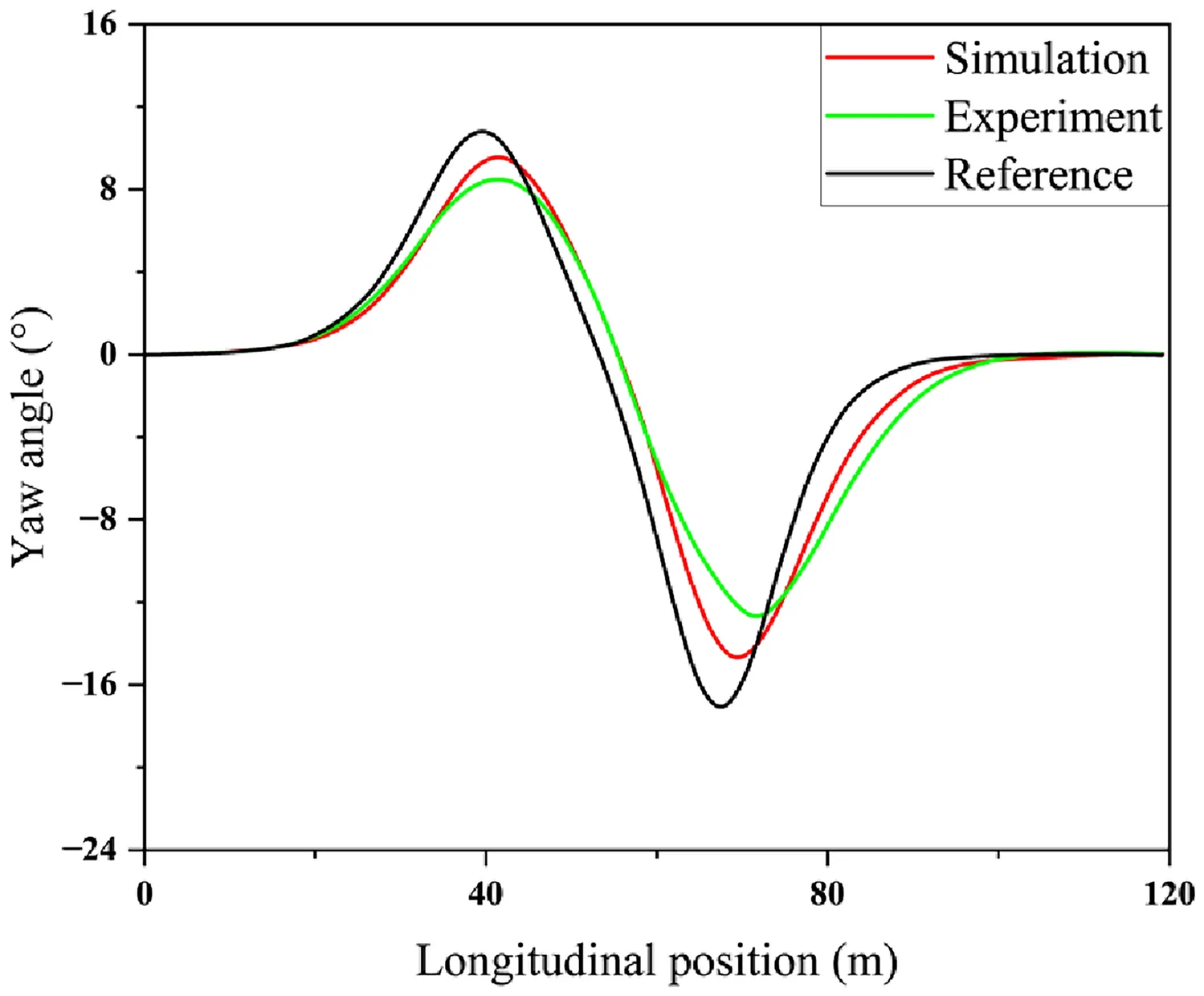
Yaw angle.

**Fig. 28:**
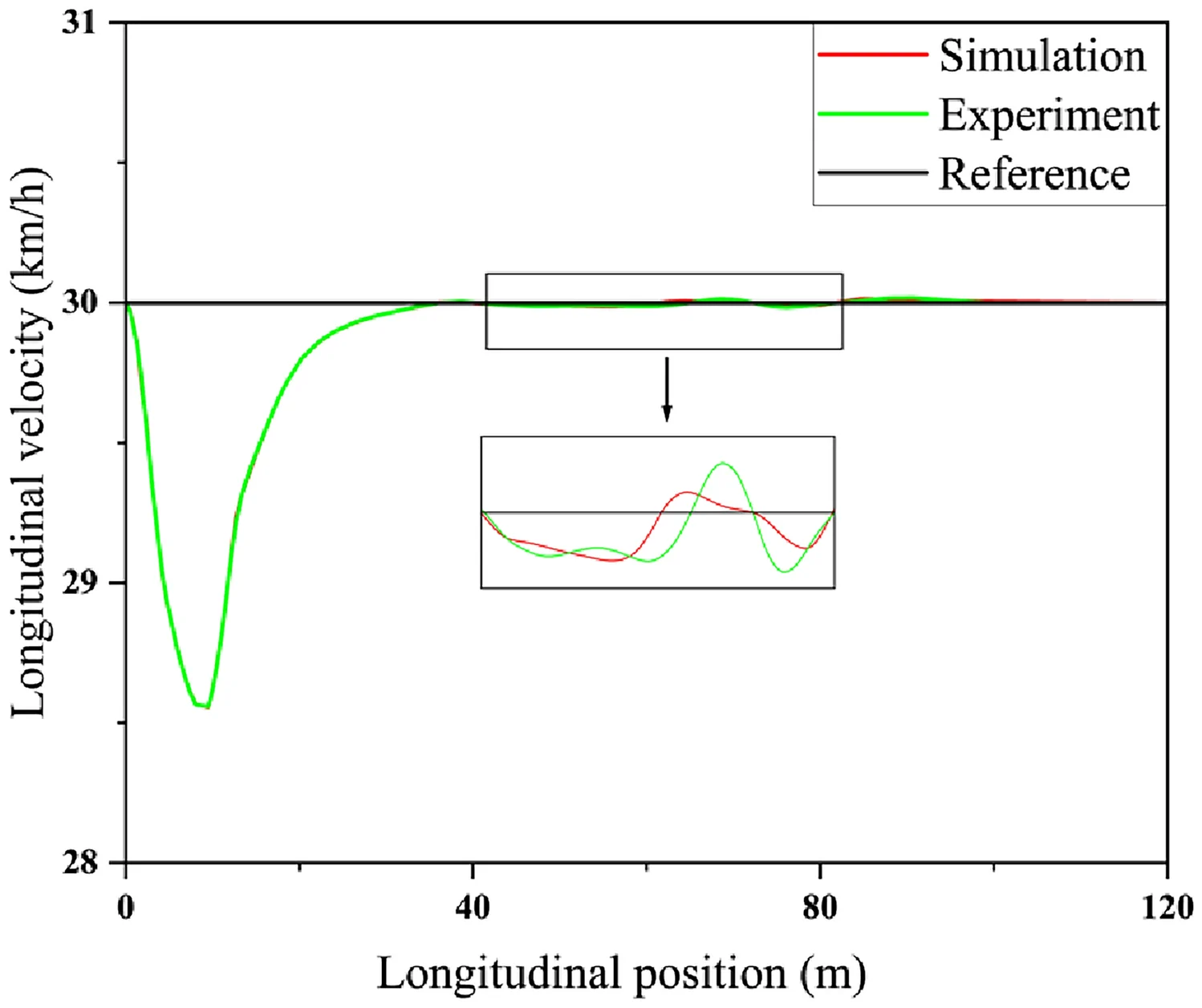
Longitudinal velocity.

**Fig. 29:**
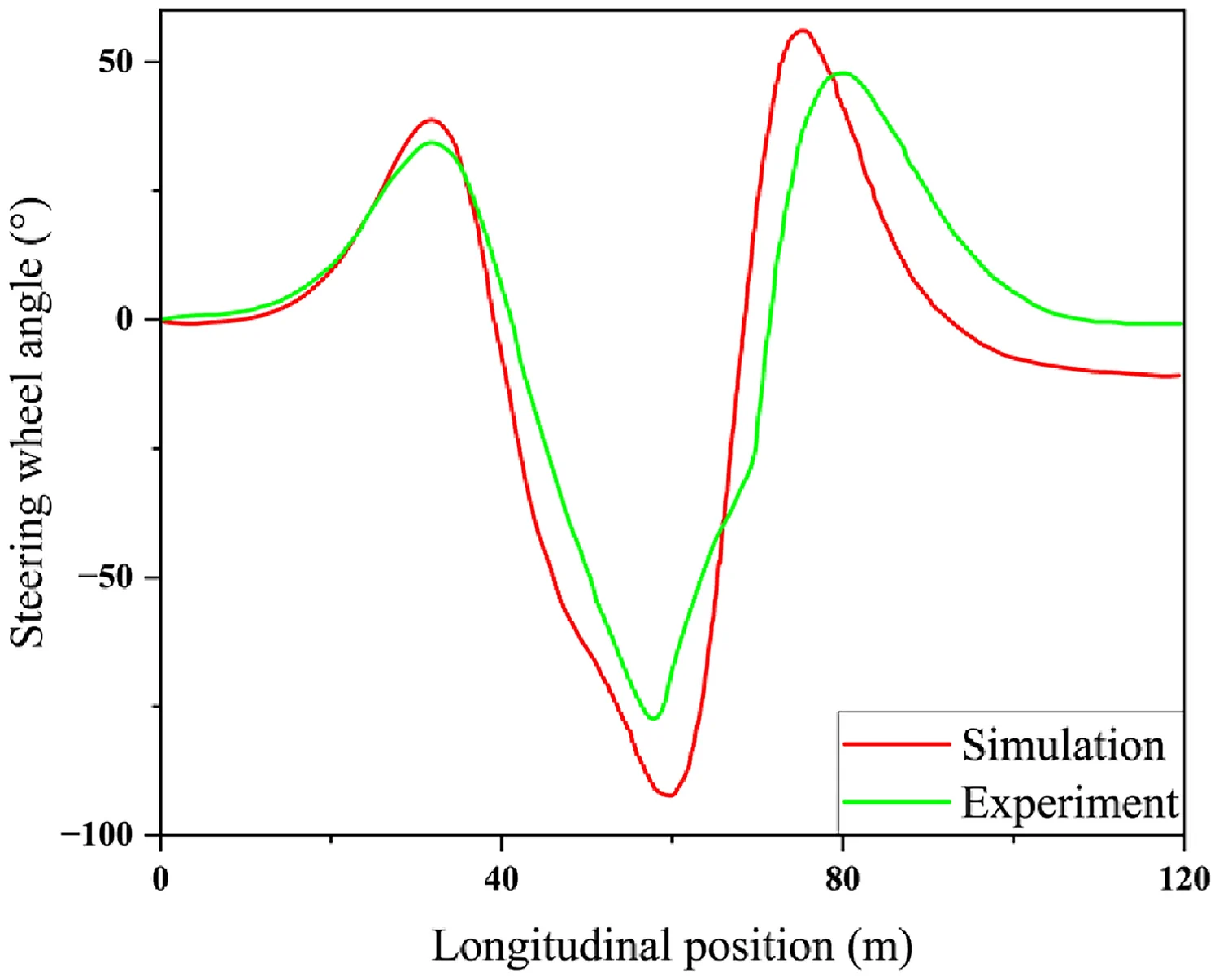
Steering wheel angle.

The vehicle travelled along the target trajectory at a constant speed of approximately 30 km/h, with its state information being acquired in real-time. Figures 26, 27 and 28 compare the HIL experimental results with offline simulation results. The recorded maximum errors were 0.56 m for lateral displacement, 6.33° for the yaw angle, and 0.05 km/h for longitudinal velocity, indicating that the experimental vehicle effectively tracked the target trajectory. Furthermore, Fig. 29 shows that the maximum error between the experimental and reference steering wheel angles was 39.99°, with both signals following largely consistent trends, further validating the reliability of the HIL experiments and the effectiveness of the control strategy.

**Fig. 30:**
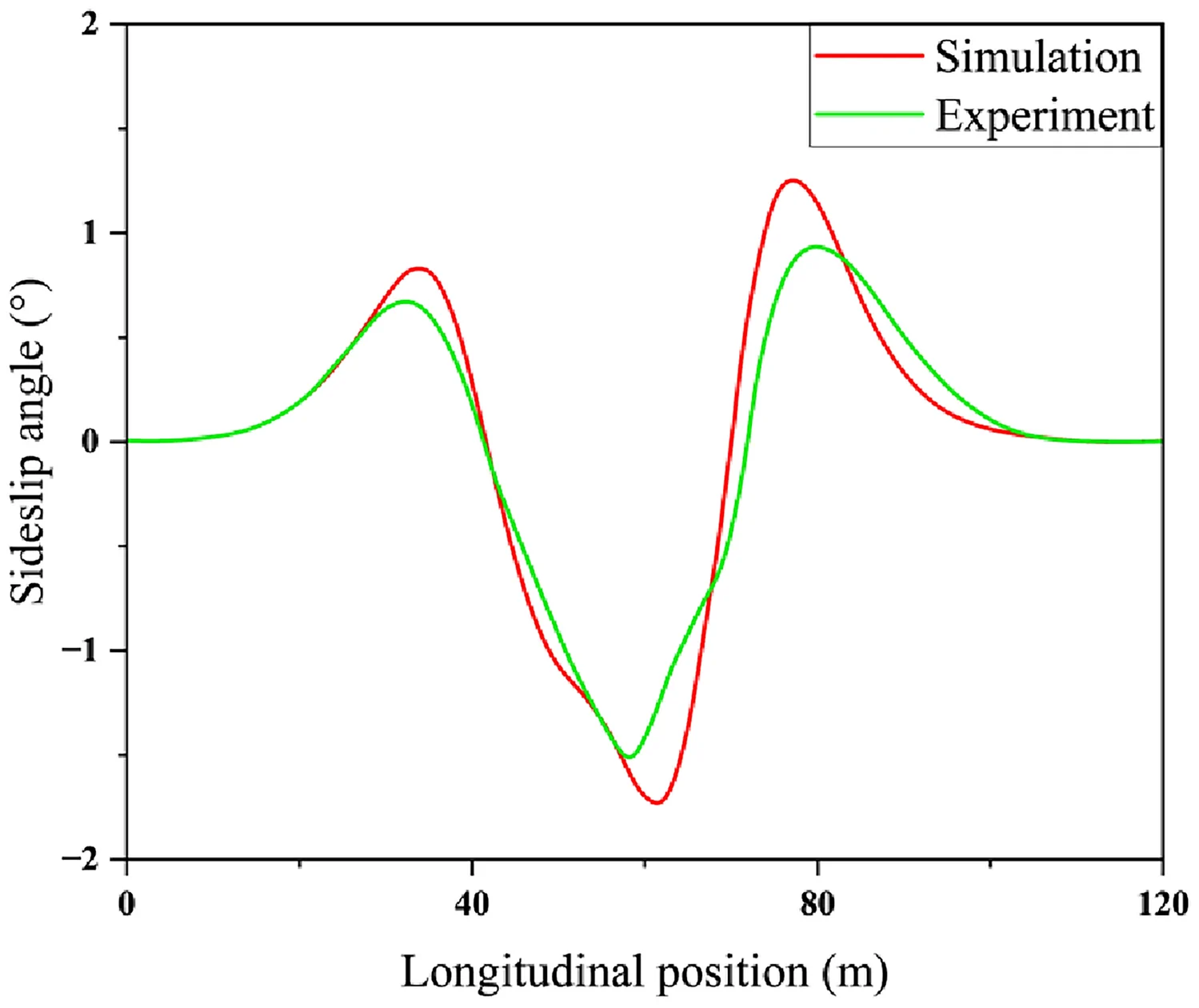
Sideslip angle.

**Fig. 31:**
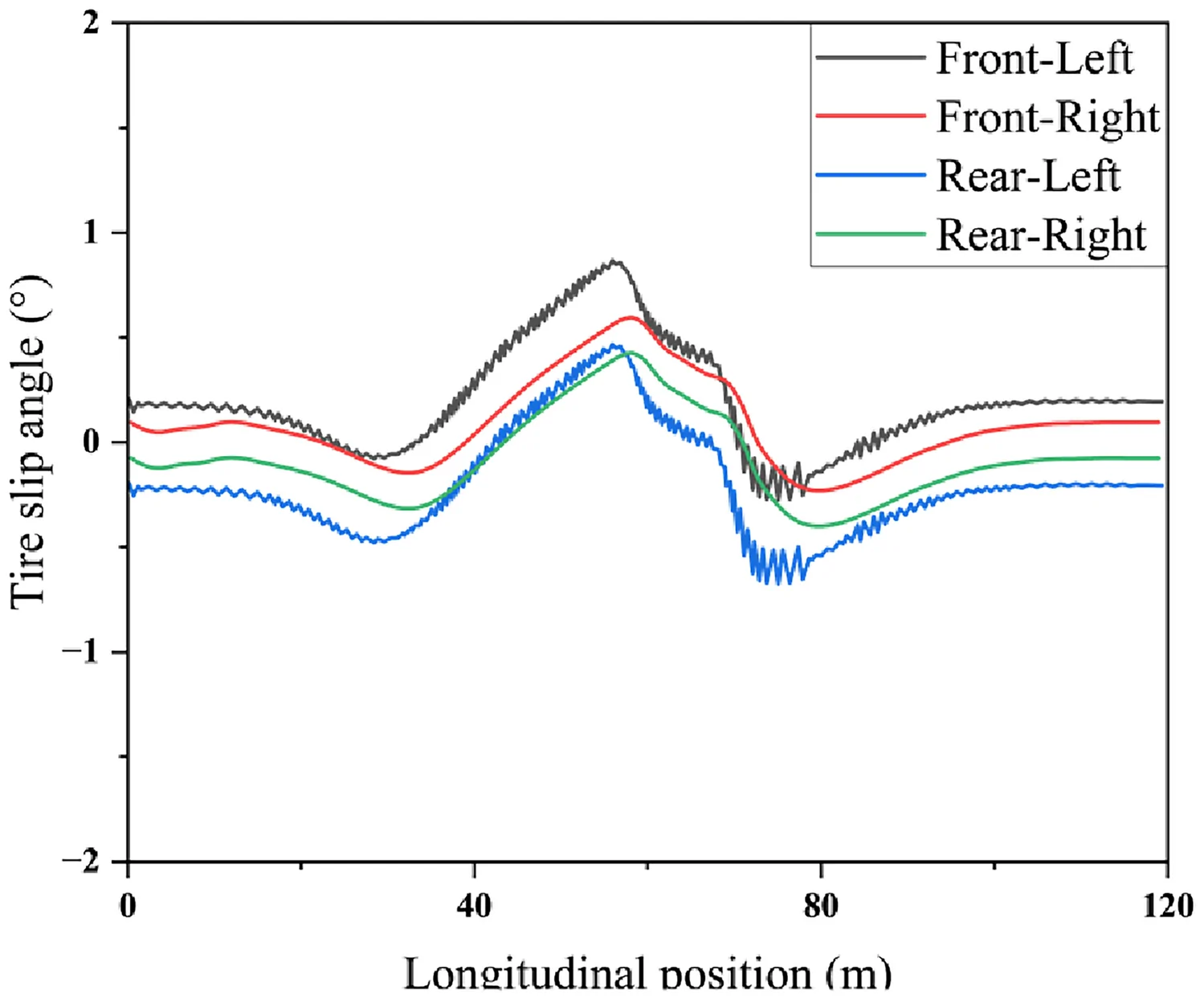
Experimental tire slip angle.

**Fig. 32:**
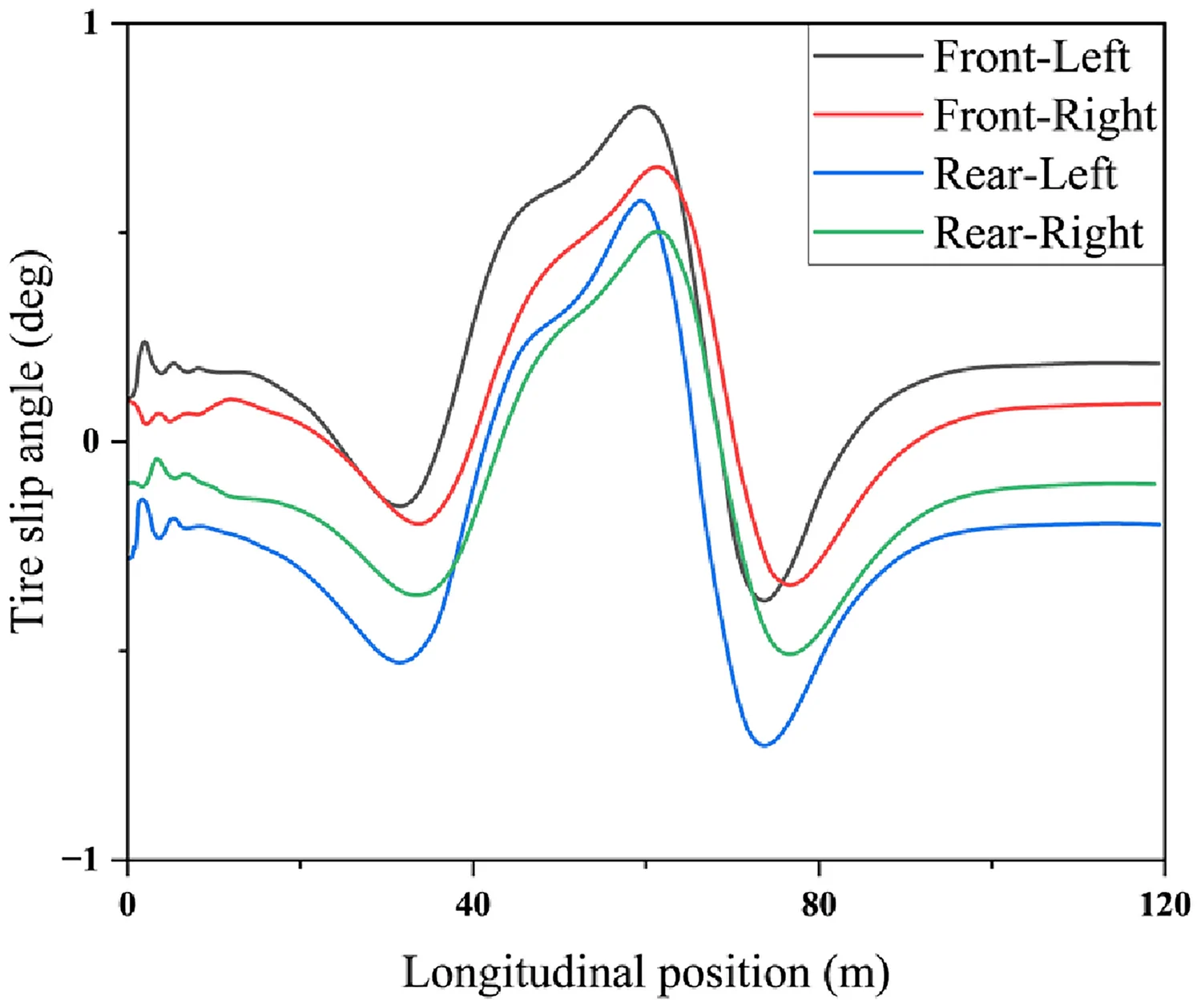
Simulated tire slip angle.

**Fig. 33:**
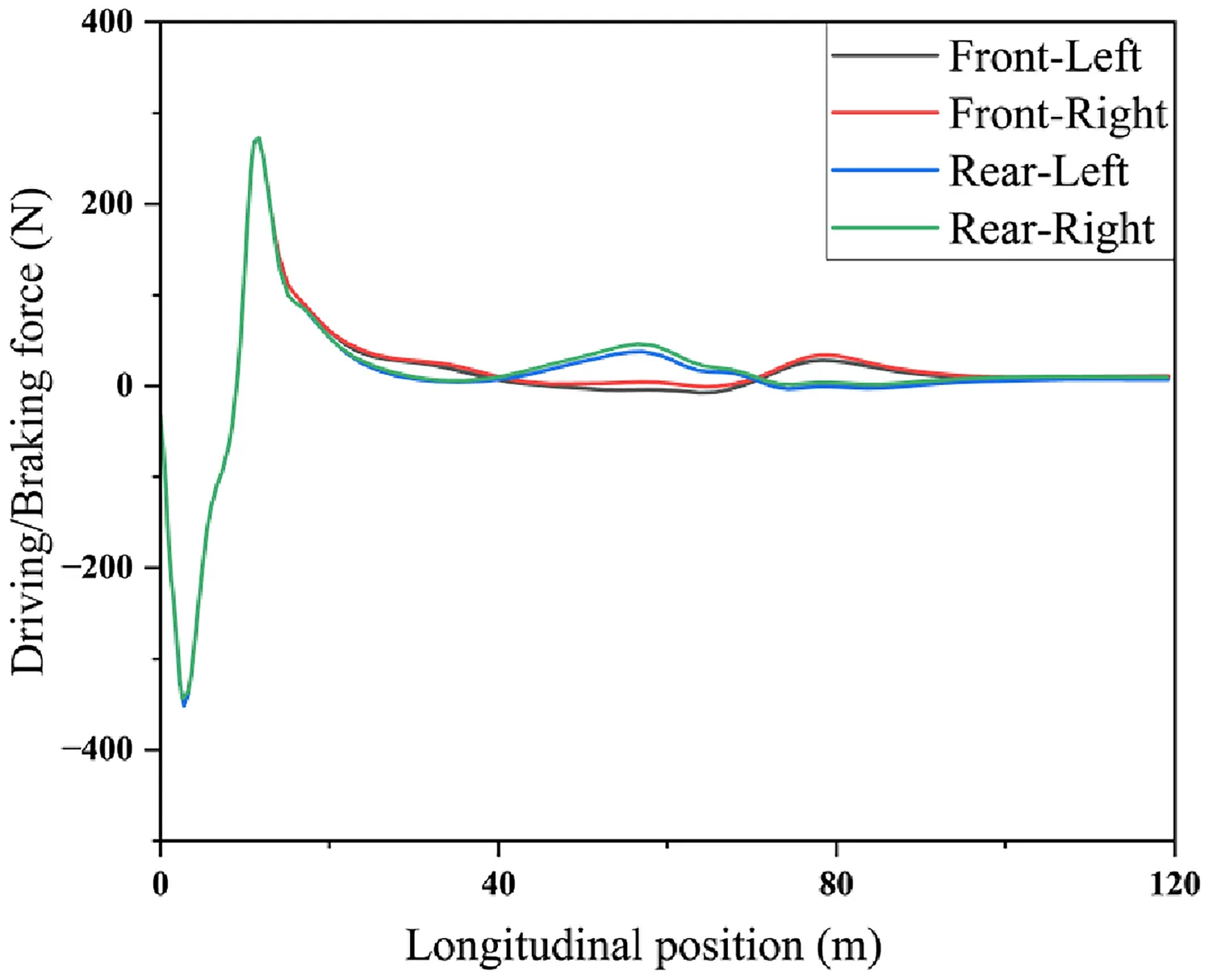
Experimental driving/braking force.

Figure 30 indicates a maximum error of 0.61° between the experimental and reference values for the sideslip angle, with both signals exhibiting good consistency in their trends. The comparative results in Figs. 31 and 32 show that some level of noise disturbance is present in the experimental data within the longitudinal displacement segment of 40–80 m. This phenomenon is likely associated with the substantial adjustments of the steering wheel angle occurring during this period. Figure 33 further presents the experimental driving/braking forces of the four wheels. It can be observed that the force distribution among the wheels remains highly consistent with the simulation trends, without noticeable actuator saturation or abnormal jitter, indicating that the proposed trajectory tracking control strategy possesses satisfactory real-time performance and executability on the actual hardware platform. Overall, the experimental data maintain a high degree of consistency with the simulation results in terms of variation trends, validating the effectiveness and real-time performance of the proposed trajectory tracking control strategy on an actual hardware platform.

### Discussion on robustness and limitations

The validation results presented in Sections "Simulation analysis" and "Simulation experimental validation of trajectory tracking control strategy" establish the effectiveness of the proposed RBF–EKF observer and LTV-MPC controller under the standard high-adhesion double lane-change benchmark. This section provides a qualitative discussion of the expected robustness of these methods to a broader range of operating conditions, based on their underlying design principles, and acknowledges the limitations of the current experimental scope.

Robustness to Road Friction Variations. As detailed in Section "MPC controller design", the stability constraint embedded in the LTV-MPC controller is an explicit function of the road friction coefficient *μ*. This analytical coupling ensures that the allowable sideslip angle envelope shrinks proportionally with decreasing friction, inherently protecting the vehicle from exceeding the reduced handling limits. Similarly, the RBF–EKF observer is trained on data encompassing nonlinear tire behavior, enabling its "pseudo-measurement" to remain informative even when the linear tire model assumption of the EKF predictor breaks down on low- *μ* surfaces.

Robustness to Sensor Noise. The EKF framework is fundamentally a minimum-variance estimator for linear(ized) systems with Gaussian noise. The recursive computation of the Kalman gain optimally weights the model prediction against the available measurements. By treating the RBF output as an additional measurement channel, the hybrid observer leverages this optimal fusion mechanism. While the current study does not include a parametric analysis of sensor noise levels, the stochastic optimality of the EKF provides a strong theoretical basis for expecting graceful performance degradation under realistic noise conditions.

Limitations and Future Work. We acknowledge that the current experimental validation is limited to a single road friction coefficient ($\mu \approx 0.9$) and does not include combined braking-steering maneuvers or a systematic variation of sensor noise intensity. These choices were made to first establish a clear baseline for the novel algorithmic contributions in a well-understood benchmark scenario. However, the absence of direct empirical evidence under these extreme conditions is a limitation of the present study. Future work will prioritize the experimental validation of the proposed integrated strategy on low-friction surfaces (e.g., $\mu \approx 0.3$ and $\mu \approx 0.5)$ and under coupled longitudinal-lateral excitation, as well as a quantitative Monte Carlo analysis of noise robustness, to further substantiate the mechanistic robustness arguments presented herein.

## Conclusion

This study systematically addressed the challenges of state estimation and trajectory tracking control for 4WID-EVs, with a focus on enhancing both tracking accuracy and dynamic stability under demanding driving conditions. The principal achievements are summarized as follows. A high-fidelity 7-DOF vehicle dynamics model was developed and validated against CarSim simulations, with results confirming its accuracy in capturing key vehicle dynamic responses. A novel hybrid observer, integrating an RBF neural network with an EKF, was proposed for vehicle state estimation. By selecting states strongly correlated with the sideslip angle as network inputs and optimizing the RBF structure, the method effectively combines the learning capability of the neural network with the noise resilience of the model-based filter. Co-simulation results demonstrated that this hybrid RBF–EKF observer significantly improves the estimation accuracy of the sideslip angle under both high-speed and low-speed, high-adhesion conditions, while also providing modest improvements in yaw rate estimation. For trajectory tracking control, an LTV-MPC strategy was designed. It employs local linearization to derive a linear predictive model and explicitly incorporates stability constraints based on the sideslip angle and tire slip angles. Simulation comparisons revealed that the proposed MPC controller outperforms the conventional PDM in tracking accuracy, control smoothness, and stability preservation. Finally, the practical viability and real-time performance of the overall strategy were successfully verified through HIL experiments on a dSPACE-based platform. Experimental results for a double lane-change maneuver showed maximum tracking errors of 0.56 m for lateral displacement, 6.33° for yaw angle, and 0.05 km/h for longitudinal velocity, with data trends consistent with offline simulations, thereby validating the effectiveness and real-time capability of the proposed integrated control strategy.

## Data Availability

Data are available on request from the authors.

## List of symbols

*a*: Distance from CoG to front axle ($\mathrm{m}$)
${a}_{x}$: Absolute longitudinal acceleration ($\mathrm{m}/{\mathrm{s}}^{2}$)
${a}_{y}$: Absolute lateral acceleration ($\mathrm{m}/{\mathrm{s}}^{2}$)
${A}_{k,t}$: System matrix of linearized model
*b*: Distance from CoG to rear axle ($\mathrm{m}$)
${B}_{k,t}$: Control matrix of linearized model
${d}_{k,t}$: Linearization error compensation term
${F}_{xij}$: Longitudinal tire force ($\mathrm{N}$)
${F}_{yij}$: Lateral tire force ($\mathrm{N}$)
${F}_{zij}$: Vertical tire load ($\mathrm{N}$)
*g*: Gravitational acceleration ($$\mathrm{m}/{\mathrm{s}}^{2}$$)
*h*: Height of vehicle center of gravity ($\mathrm{m}$)
${H}_{p}$: Prediction horizon of MPC
${H}_{c}$: Control horizon of MPC
*I*: Identity matrix
${I}_{\omega }$: Wheel rotational inertia ($\mathrm{kg}\,{\mathrm{m}}^{2}$)
${I}_{zz}$: Yaw moment of inertia of vehicle ($\mathrm{kg}\,{\mathrm{m}}^{2}$)
${k}_{1}$: Front axle tire cornering stiffness ($\mathrm{N}/\mathrm{rad}$)
${k}_{2}$: Rear axle tire cornering stiffness ($\mathrm{N}/\mathrm{rad}$)
${l}_{f}$: Front track width ($\mathrm{m}$)
${l}_{r}$: Rear track width ($\mathrm{m}$)
*L*: Wheelbase (L = a + b) ($$\mathrm{m}$$)
*m*: Total vehicle mass ($\mathrm{k}\mathrm{g}$)
${M}_{z}$: External yaw moment ($\mathrm{N}\,\mathrm{m}$)
*Q*: Weight matrix of state error
*R*: Weight matrix of control increment
*r*: Yaw rate ($$\mathrm{rad}/\mathrm{s}$$)
${R}_{e}$: Effective rolling radius of tire ($\mathrm{m}$)
*s*: Complex frequency in Laplace domain
*T*: Sampling time ($$\mathrm{s}$$)
${T}_{a}$: Actual electromagnetic torque ($\mathrm{N}\,\mathrm{m}$)
${T}_{d,ij}$: Wheel driving torque ($\mathrm{N}\,\mathrm{m}$)
${T}_{b,ij}$: Wheel braking torque ($\mathrm{N}\,\mathrm{m}$)
${T}_{x,ij}$: Ground reaction torque ($\mathrm{N}\,\mathrm{m}$)
*u*: Control input vector
*Δ u*: Control increment vector
${v}_{x}$: Longitudinal velocity at CoG ($\mathrm{m}/\mathrm{s}$)
${v}_{y}$: Lateral velocity at CoG ($\mathrm{m}/\mathrm{s}$)
${v}_{xij}$: Longitudinal velocity of wheel center ($\mathrm{m}/\mathrm{s}$)
*X*: System state vector
*Y/X*: Lateral/longitudinal position in global frame ($\mathrm{m}$)
${\alpha}_{ij}$: Tire slip angle ($\mathrm{rad}$)
*β*: Vehicle sideslip angle at CoG ($\mathrm{rad}$)
*δ*: Front wheel steering angle ($\mathrm{rad}$)
${\delta}_{f}$: Front wheel steering angle ($\mathrm{rad}$)
${\delta}_{s}$: Steering wheel angle ($\mathrm{rad}$)
*ε*: Slack variable of MPC
*η*: Output vector of system
${\lambda}_{ij}$: Wheel slip ratio
*μ*: Road friction coefficient
*ξ*: Damping ratio of motor
*ρ*: Weight coefficient of slack variable
*ϕ*: Yaw angle ($$\mathrm{rad}$$)
${\omega}_{ij}$: Wheel rotational speed ($\mathrm{rad}/\mathrm{s}$)
*fl*: Front-left wheel
*fr*: Front-right wheel
*rl*: Rear-left wheel
*rr*: Rear-right wheel
